# Prevalence and risk factors for key infectious diseases amongst migrants to the UK: a systematic review

**DOI:** 10.1186/s12879-026-12953-z

**Published:** 2026-03-03

**Authors:** Rebecca F. Baggaley, Carys M. Hooper, Luisa Silva, Zainab Lal, Paul Bird, Dee Menezes, Dominik Zenner, Christopher A. Martin, Manish Pareek

**Affiliations:** 1 Institute of Health Informatics, University College London, 222 Euston Road, London, NW1 2DA UK; 2 Division of Public Health and Epidemiology, School of Medical Sciences, University of Leicester, Leicester, UK; 3 Development Centre for Population Health, University of Leicester, Leicester, UK; 4 Infection and Immunity Research Unit, St George’s University of London, London, UK; 5 Department of Clinical Microbiology, University Hospitals of Leicester NHS Trust, Leicester, UK; 6 Wolfson Institute of Population Health, Queen Mary University of London, London, UK; 7 Department of Infection and HIV Medicine, University Hospitals of Leicester NHS Trust, Leicester, UK; 8 NIHR Applied Research Collaboration East Midlands, University of Leicester, Leicester, UK; 9 NIHR Leicester Biomedical Research Centre (BRC), University of Leicester, Leicester, UK; 10 Queen Mary and Barts Health Tuberculosis Centre, Blizard Institute, Queen Mary University of London, London, UK

**Keywords:** Migrant, Refugee, Asylum seeker, HIV, Tuberculosis, Viral hepatitis, HBV, HCV, Infection prevalence

## Abstract

**Background:**

Migrants are at increased risk of infections including HIV, tuberculosis and viral hepatitis, with poorer outcomes. Early diagnosis and management can reduce morbidity, mortality and onward transmission. This systematic review summarises prevalence of HIV, latent and active tuberculosis and hepatitis B and C among UK migrants and evaluates associated risk factors.

**Methods:**

PubMed/Medline, EMBASE, Web of Science and the Cochrane Library were systematically searched from 2004 to 11 June 2025. The review was conducted using PRISMA guidelines and registered with PROSPERO (registration CRD42024521191). Quality assessment was performed using the Joanna Briggs Institute Critical Appraisal Checklist for Prevalence Studies. High heterogeneity (I^2^ = 95.2%, 99.2%, 87.2%, 96.9% and 91.6% for IGRA, active TB, HIV, HBV and HCV yields, respectively) indicated that meta-analysis was not appropriate. The impact of risk factors on prevalence was explored through meta-regression and descriptive analysis.

**Results:**

Of 2033 identified records, 36 were included, reporting Interferon Gamma Release Assay (IGRA) (*n* = 13), active TB (*n* = 10), HIV (*n* = 12), HBV (*n* = 16) and HCV (*n* = 11) test yields. An additional two publications excluded from the main analysis for reporting duplicate study data were included in the risk factor analysis because they stratified prevalence by additional risk factors. Highest yield was for IGRA which, excluding one lower prevalence outlier (6.9% (*n* = 1617)), was 15.1%–22.1%. There was high heterogeneity in active TB prevalence: 62–1,484/100,000. HIV prevalence among larger studies (*n* > 200) was 0.18%–0.48%. HBV prevalence was 0.00%–8.93% (all studies) and 1.06%–4.75% for larger studies (*n* > 1000). HCV prevalence was lower: 0.00%–1.67%, with only two of 11 included estimates above 0.50%. There was considerable heterogeneity in risk factors analysed making comparisons difficult.

**Conclusions:**

Despite heterogeneity, infection prevalence was generally high, particularly IGRA yield and HBV. This underscores the need to maintain effective monitoring, testing and treatment for key infections among migrant populations, especially given the rapidly evolving epidemiological and demographic landscape for this population.

**Supplementary Information:**

The online version contains supplementary material available at 10.1186/s12879-026-12953-z.

## Introduction

Migrants to the UK are at increased risk of communicable diseases compared to the general population, and the chronic infections tuberculosis (TB) and blood-borne viruses (BBVs) HIV, hepatitis B, and hepatitis C have been identified as key health priorities for migrants by international and national organisations, advocating an integrated approach to testing [1–3]. In 2024, 82% of TB notifications were in people born outside the UK [4]. An international systematic review, including UK-based studies, has shown that HIV prevalence is higher in migrants than in native-born populations [5]. The United Kingdom Health Security Agency (UKHSA) reported in 2024 that 95% of new chronic hepatitis B diagnoses in the UK are in migrants [6], and HCV seroprevalence in women delivering live-born infants in North Thames, England is higher in non-UK-born than UK-born mothers [7].

Increased infection risk stems from multiple factors across the migration journey – before, during and after arrival – including greater exposure to infections, substandard living conditions, and limited access to healthcare [8]. Evidence shows that migrants are more likely to present later with these infections (e.g., overseas-born individuals are significantly more likely to present with HIV infection at CD4 counts below 350 cells/mm³ [9]). They may also experience more aggressive disease progression, as seen with TB [10], and may transmit infections to contacts if left undiagnosed [11, 12].

Early diagnosis and management of these infections are crucial for improving health outcomes by reducing morbidity, mortality and onward transmission [13]. This position is supported by several national guidelines, including those from the National Institute for Health and Care Excellence (NICE), which recommend screening migrants for active and latent TB [14, 15], HIV [16–18], hepatitis B [19, 20] and hepatitis C [19–21] to support national prevention targets [19, 22–24].

In many low TB incidence countries (World Health Organization definition of < 10 per 100,000 population [25]) such as the UK, as well as countries in the European Union, North America, Australia and New Zealand, most active TB cases in migrants result from reactivation of latent TB infection [26]. The stressors associated with migration may act as a trigger for this reactivation, underscoring the importance of screening and treating both latent and active infections [27]. Furthermore, TB prevention and control are particularly important now, given that TB notifications are increasing in the UK [28].

As we attempt to reach targets for control of these infections, we need to know prevalence of infection within specific risk groups such as migrants for effective health planning, particularly for approaches to screening. In this systematic review, we aimed to evaluate recent (within the past 21 years) evidence regarding prevalence of key infections (HIV, TB and viral hepatitis) amongst migrants to the UK, to aid planning appropriate, effective interventions to screen migrant populations. We also aimed to explore prevalence stratified by established and potential risk factors for infection, which could inform targeted testing strategies adopting a risk-based approach.

## Methods

This systematic review was conducted according to the Preferred Reporting Items for Systematic Reviews and Meta-Analyses (PRISMA) guidelines [29] to uphold the integrity, transparency and reproducibility of the research process. The study was registered with PROSPERO (CRD42024521191). The primary outcomes of interest were point prevalence and/or test yield for active TB, HIV, HBV, HCV and IGRA test positivity (used as a proxy for latent TB infection or exposure to TB infection). Secondary outcomes were primary outcomes stratified by risk factors, where reported. We did not predefine these risk factors but extracted data for all factors reported by included studies.

### Search strategy

We searched PubMed/Medline, EMBASE, Web of Science and the Cochrane Library from 2004 to 11 June 2025 for studies reporting prevalence of any one or more of the included infections (HIV, TB, HBV and HCV) among any international migrant type migrating to the UK. The search conducted up to March 2024 included publications from the preceding 20 years, as data from earlier periods were considered less relevant to current infection prevalence. A 20 year-window was considered sufficient to yield an adequate number of studies for each infection and to facilitate the examination of temporal trends. This original search was subsequently updated in March and June 2025, resulting in a total 21 years of data being included. There were no language or publication status restrictions. We used the following Medical Subject Heading (MeSH) and keyword terms: (HIV OR tuberculosis OR TB OR hepatitis B OR HBV OR hepatitis C OR HCV OR “viral hepatitis”) AND (migra* OR immigra* OR refugee* OR asylum) AND prevalence AND (England OR Scotland OR Wales OR Ireland OR Britain OR United Kingdom OR UK).

### Eligibility criteria

Studies were included if they reported prevalence of HIV, HBV, HCV or active TB infection, or test positivity rates (test yields) for these infections, including IGRA testing for latent TB infection/past exposure (or from which prevalence could be derived), for any international migrant type settling or settled in the UK. We used the International Organization for Migration (IOM) definition of migrant [30]. Estimates were also included where the study population, or a subgroup of the study population, was described as non-UK-born and where there was clear implication that these study participants were residing in the UK long-term. Studies reporting results from pre-entry migrant screening programmes were included. Exclusion criteria included estimates based on self-reported diagnoses, prevalence reported for travellers/international exchange students rather than migrants, case reports, contact tracing studies, outbreak investigations, editorials and reviews. Modelling studies, where the denominator was an estimate of the migrant population size rather than representing all migrants who were tested for/clinically examined to diagnose infection, were excluded. Studies which only reported prevalence of specific TB conditions (e.g., pulmonary, extrapulmonary, spinal TB) were also excluded. Studies were excluded if the data they reported were superseded by more recent studies, unless the earlier study reported more relevant information. For the secondary outcomes (prevalence/test yields stratified by risk factors), estimates of relative risk were insufficient for inclusion – number tested and number diagnosed/testing positive had to be stated. There was no restriction based on age, sex or ethnicity.

### Study selection

Records identified through database searching were merged and duplicates removed using Rayyan Software [31]. Two reviewers (CMH, LS, LZ, PB), including the lead investigator (RFB), independently screened the records by title/abstract and full text according to the inclusion/exclusion criteria. Any disagreements were resolved by the lead investigator.

### Data extraction

Data were extracted using a predefined form to capture study information including author, year of publication, study characteristics, study participant demographics, test type, numbers tested and test outcomes, including coinfection estimates. While we only included prevalence of active HCV infection in the analysis, we also recorded estimates of HCV antibody test positivity, indicating previous history of HCV infection. Where reported, we included test outcomes stratified by patient characteristics/risk factors. The data extraction form was refined during the extraction of the first few articles to ensure the forms were comprehensive. Two reviewers (RFB with CMH, LS, LZ or PB) independently extracted data, with discrepancies resolved through consensus. We contacted eight authors for further information; seven replied and one provided further data.

### Quality assessment

All studies were assessed independently by two reviewers (RFB with CMH, LS, LZ, or PB) using the Joanna Briggs Institute Critical Appraisal Checklist for Prevalence Studies [32, 33] to identify potential biases and determine the quality of evidence the studies provide, and were categorised as being of high, moderate or low quality. Discrepancies were resolved through consensus.

### Data synthesis and analysis

We summarised prevalence data from included studies using forest plots (produced in R version 4.4.2 using the *meta* package), stratified by infection type. All prevalence estimates and 95% confidence intervals (95%CI) were recalculated using data from each study on numbers tested and numbers testing positive, to ensure consistency (95%CI were calculated using the Wilson score method). We evaluated heterogeneity in prevalence estimates using the I^2^ statistic and, given the high level of heterogeneity observed for all infections (I^2^ >75% is assumed to represent considerable heterogeneity) and the variability in study populations, pooling of estimates through formal meta-analysis was not considered to be appropriate. Instead, qualitative synthesis was conducted to describe and summarise findings and identify patterns across studies.

For HIV, estimates were categorised as extremely high (> 0.5%), high (0.2–0.5%) and low (< 0.2%) prevalence, based on established thresholds used to define UK local authorities [34, 35]. HBV estimates were categorised as high (≥ 5%), intermediate (2-4.9%) and low (< 2%) prevalence to align with current World Health Organization national endemicity categories [36]. In the UK, HCV prevalence is not typically defined using formal numerical categories. We therefore defined a pragmatic, informal categorisation of prevalence, for descriptive purposes only, using the 0.12% estimated prevalence for the general population in England in 2023 [37], classing up to 150% of general population prevalence as low (up to 0.18%), up to three times the general population prevalence as intermediate (> 0.18% up to 0.36%) and above that as high (> 0.36%) prevalence. A similar approach was applied for IGRA yield, because IGRA testing has been restricted to targeted populations rather than broad, unselected population surveys. A hospital-based TB outbreak study reported an IGRA yield of 8.7% among unexposed age- and other demographics-matched control patients [38], which may overestimate yield in the general population, as patients may experience more exposures. In contrast, a study of farm workers in England with potential *Mycobacterium bovis* exposure reported an overall IGRA positivity rate of only 1.1%, despite occupational exposure (but likely few other risk factors for exposure) [39]. We therefore assumed a UK general population IGRA positivity rate of 5% to inform categories, using the same methodology as for HCV: classing up to 150% of general population IGRA positivity as low (up to 7.5%), up to three times as intermediate (> 7.5% to 15%) and above that as high (> 15%) test positivity. In the absence of formal prevalence categories for active TB, we used migrant country-of-origin TB incidence screening thresholds as a pragmatic, informal indicator. High prevalence was defined as ≥ 40 per 100,000 (the threshold used for UK pre-entry active TB screening [40]), and extremely high prevalence as ≥ 150 per 100,000 (the threshold used for UK latent TB infection testing [40]).

We then conducted a risk factor analysis, which included conducting univariable and multivariable meta-regression analyses for each infection, using continuous covariates (median date of study data collection) and covariates that could be categorised into a limited number of groups: study quality (high, moderate, low) and timing of testing (pre-entry, at entry or post arrival to the UK). Number of covariates included in multivariable meta-regression was restricted to these, to reflect the limited number of study estimates for each infection and the incomplete data reported by studies for many potential covariates. Characteristics of migrant populations (migrant type e.g., asylum seekers, students, sexual health clinic attendees etc.) reported by studies were very diverse, and so they were summarised in text form on forest plots, for descriptive analysis. We attempted visually to synthesise estimates stratified by age, sex and country of birth/origin of migrants using heat maps, aligning mismatched risk factor categories (e.g., different age group definitions between studies) as far as possible and using the infection prevalence categories described above. Spatial visualisations were produced in R using the *tmap* package to create choropleth maps showing the distribution of studies across UK counties and unitary authorities using Office for National Statistics 2023 data [41]. In addition, we compared infection prevalence estimates for UK migrants stratified by country of origin with prevalence in country of origin, using data for HIV [42], HBV and HCV [43] (there were insufficient comparable data to perform a comparison for active TB and IGRA yield). Results were summarised as scatter plots.

## Results

**Fig. 1:**
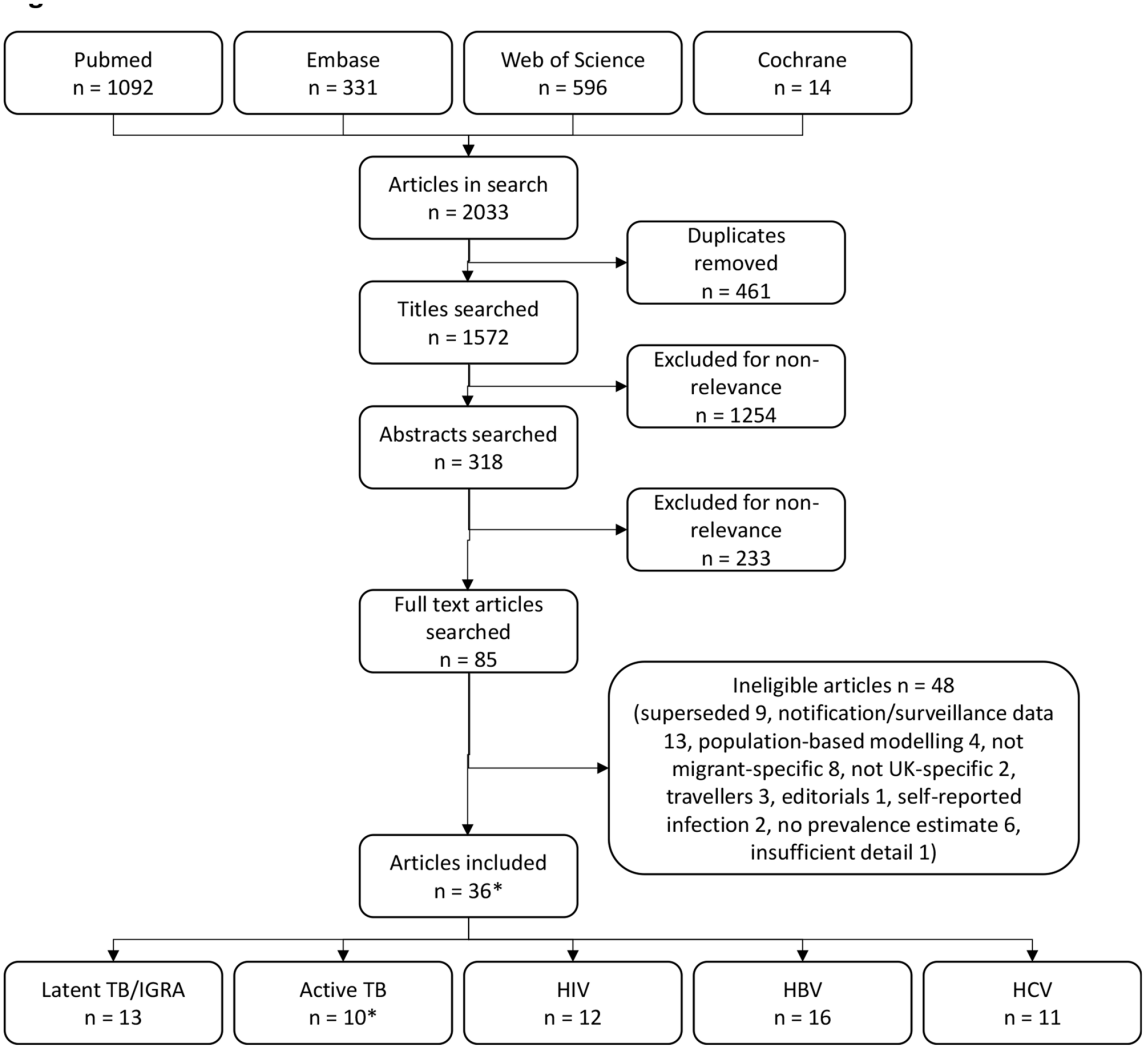
Preferred Reporting Items for Systematic Reviews and Meta-Analyses (PRISMA) flow diagram of included studies. *Two studies reporting prevalence of active TB are not included in the forest plot analysis because of overlapping data: overall prevalence estimates are reported by Menezes et al. [45], but Aldridge et al. [56] and Zenner et al. [57] both provide additional prevalence data for the same study population stratified by additional risk factors and have been included in the risk factor analysis

The database search yielded 2033 articles. 1572 articles were unique, after removal of duplicates, and of these, 85 were selected for full-text screening. Review of the full-text articles led to the inclusion of 36 articles in the analysis (see PRISMA flow diagram, Fig. 1). Included studies reported prevalence of latent TB infection/IGRA positivity (*n* = 13), and active TB (*n* = 10), HIV (*n* = 12), HBV (*n* = 16) and HCV (*n* = 11) infection. Two studies reported prevalence from pre-entry screening. Crawshaw et al. reported testing data from International Organisation for Migration clinics enrolled in the UK pre-entry migration health assessments, for refugees resettled in the UK [44]. Menezes et al. reported data extracted from the UK new entrant screening programme (which does not include asylum seekers, who undergo domestic health checks and are not part of pre-entry screening) to compare infection prevalence with other European countries [45]. The remaining studies were conducted at entry to the UK (defined as immediately after arrival e.g., testing at airports [46] or shortly afterwards e.g., asylum seekers at initial accommodation centres [47]); or post-arrival (after the at entry stage e.g., testing upon registration with primary care [48]).

More studies were conducted in London than elsewhere in the UK (*n* = 23 in London or Greater London, including studies with catchment defined as England or the UK) (see map of distribution of study settings, Supplementary Material Fig. S1). The next most frequent study location was Birmingham (10 studies). Two studies were classified as UK-based because they reported results of UK pre-entry screening [44, 45]; one was a national screening programme for Ukrainian refugees in Wales [49] and five were based on testing migrants across England [50–54]. The remaining studies had smaller study regions but often included multiple study sites; for example, Flanagan et al. reported from areas of the UK with a high density of migrants (Bradford, Yorkshire, and northeast and southeast London) [55]. However, these studies did not usually report prevalence estimates stratified by study region, and the limited number of included studies makes comparisons between studies by UK study region difficult.

**Fig. 2:**
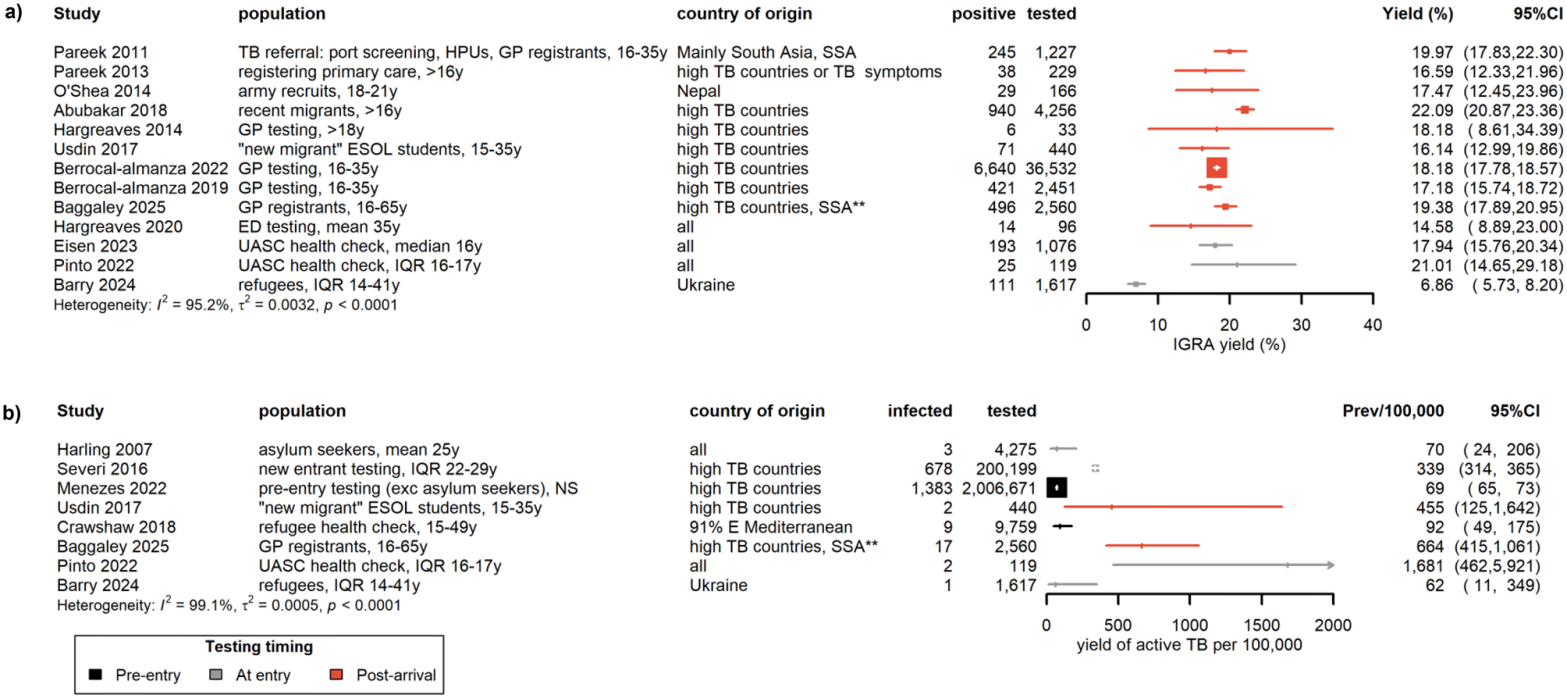
Forest plots of prevalence estimates for a) IGRA yield (%) and **b**) prevalence of active TB (yield per 100,000). Estimates are listed from oldest to most recent based on the median year of data collection. * Overseas-born individuals 16–65y, UK arrival < 5 years, from a high TB incidence country (≥ 150/100,000) or from sub-Saharan Africa or a refugee/asylum seeker (IGRA testing for patients ≤ 35 years only) [48]. Only patients testing IGRA positive were examined for signs of active TB. ** Asylum seekers in the UK undergo domestic health checks and are not part of pre-entry screening. Menezes et al. reported that the majority of screening episodes were among persons with student (45%) or settlement visas (24%), with lower proportions among those on work visas (8%), family reunification (4%) and working holiday maker visas (2%) [45]. E – East; ED – Emergency Department; ESOL – English for Speaker of Other Languages course; exc – excluding; GP – general practice and primary care; HPU – Health Protection Unit; IQR – interquartile range; NS – not stated; SSA – sub Saharan Africa; UASC – unaccompanied asylum seeker children; y – years

**Fig. 3:**
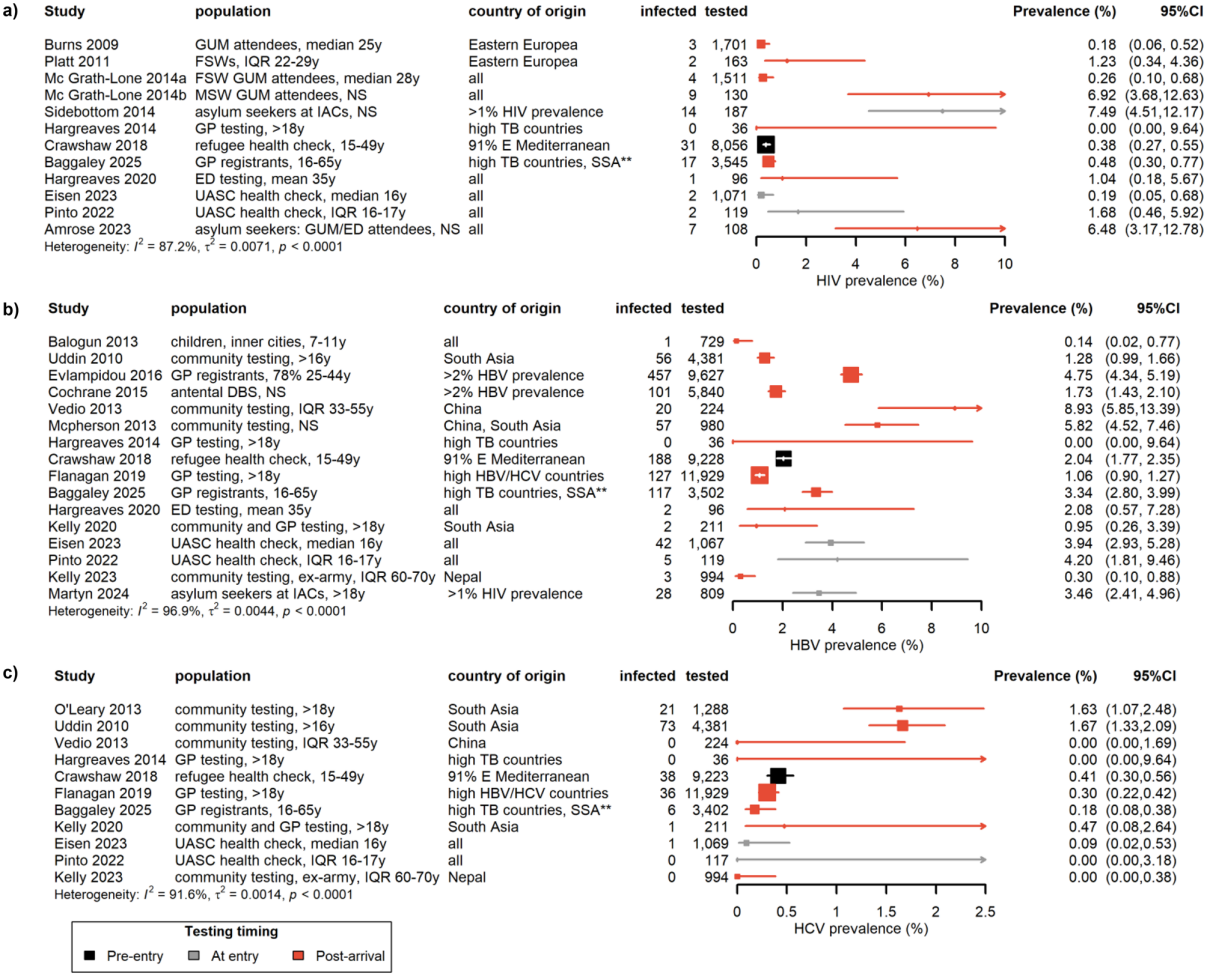
Forest plots of % prevalence estimates for a) HIV, b) HBV and **c**) HCV. Estimates are listed from oldest to most recent based on the median year of data collection. * Overseas-born individuals 16–65y, UK arrival < 5 years, from a high TB incidence country (≥ 150/100,000) or from sub-Saharan Africa or a refugee/asylum seeker (IGRA testing for patients ≤ 35 years only) [48]. DBS – neonatal dried blood spot testing; ED – Emergency Department; FSW – female sex worker; GP – general practice and primary care; GUM – genitourinary medicine or sexual health clinic; IAC – Initial Accommodation Centre for UK asylum seekers; MSW – male sex worker; NS – not stated; UASC – unaccompanied asylum seeker children; y – years

Two of the ten studies reporting prevalence of active TB [56, 57] were not included in the forest plot analysis because Menezes et al. [45] reported prevalence from the same study population, but these studies were included in the analysis of prevalence stratified by risk factors because they provided additional data. Therefore the 34 articles reporting on different study samples shown across the forest summary plots (Figs. 2 and 3) included a total of 2,313,965 migrants tested. A detailed summary of all included studies is presented in Table S1, Supplementary Material. Table S2 lists studies that were excluded because they reported prevalence of HCV antibody test positivity only (indicating prevalence of history of HCV infection rather than current, active infection).

### Study quality

Table S3, Supplementary Material, reports the quality assessment of included studies using the Joanna Briggs Quality Assessment Tool [32]. A large majority of included studies were of high quality. In total, 25 studies were appraised as high, seven as moderate and four as low quality (10 of 13 IGRA yield estimates were from high quality studies and similarly, 5 of 8, 6 of 12, 10 of 16, and 8 of 11 estimates for active TB, HIV, HBV and HCV prevalence were from high quality studies, respectively). Three low quality studies were conference abstracts, which necessarily are limited in the level of detail that can be reported.

### Overall prevalence estimates

Figure 2 summarises IGRA yield and infection prevalence estimates for active TB, while Fig. 3 summarises infection prevalence estimates for HIV, HBV and HCV. As anticipated, given the heterogeneity in included study populations, the I^2^ statistic for each infection indicated substantial heterogeneity (95.2% for IGRA yield and 99.1%, 87.2%, 96.9%, and 91.6% for prevalence of active TB, HIV, HBV and HCV, respectively). We therefore did not conduct a meta-analysis. Study populations varied from a broad inclusion of migrants (e.g., migrants registering with primary care [48, 58]) to specific populations (e.g., army recruits among Nepalese new arrivals [51]; results from Unaccompanied Asylum Seeking Children (UASC) health checks [59]; Ukrainian refugees [49]; Eastern European Genitourinary Medicine (GUM) clinic attendees [60]), making comparisons between the limited number of studies difficult.

The highest prevalence values were observed for IGRA yield where, excluding one outlier with lower prevalence (6.86%, 95%CI 5.73–8.20%, *n* = 1617, Ukrainian refugees [49]), central estimates of yield were relatively homogeneous, varying between 14.58% (95%CI 8.89-23.00%) and 22.09% (95%CI 20.87–23.36%) (*n* = 12) with no suggestion of a change in yield over time.

There was high heterogeneity for active TB studies, both in terms of prevalence estimates and study sample sizes (which ranged from 119 [49] to more than two million [45]). Extremely high prevalence was reported from studies screening UASC (Pinto et al. abstract, diagnostic method not stated: 1,681/100,000, 95%CI 462-5,921, *n* = 119 [61]) and those restricting screening to individuals from high TB incidence countries (recent migrants: 1484/100,000, 95%CI 1083–2031 [48]; 455/100,000, 95%CI 125-1,642 [62]; and new entrants: 339/100,000, 95%CI 314–365 [46]). The extremely high prevalence reported by Baggaley et al. reflects a study in which active TB was diagnosed among IGRA-positive migrants, supported by detailed clinical, biological, and radiological assessment—an approach likely to have enhanced diagnostic sensitivity [48]. In contrast, Menezes et al. reported a much lower (though still high according to our pragmatic, informal indicator) prevalence of 69/100,000 (95%CI 65–73) for the 2,006,671 individuals undertaking pre-entry screening in their country of origin by designated clinics [45], although again, these individuals undergoing pre-entry screening were from high TB incidence countries (> 40 per 100,000 population [45, 56]). Earlier studies, including Menezes et al., involved use of less sensitive diagnostic methods such as symptom-based and/or chest X-ray. The two more recent (2018 onwards) published studies which reported relatively low prevalence estimates, despite using more detailed assessment for diagnosis [44, 49], included migrants who were likely to be at lower risk of infection because their countries of origin have lower TB prevalence (primarily Eastern Mediterranean countries [44]; Ukraine [49]) than the countries of origin of migrants in the other recent studies (sub Saharan Africa, high TB incidence countries in Asia) [63].

Sample sizes tended to be smaller for studies reporting HIV prevalence. HIV prevalence estimates were generally high compared to the UK general population (nine of 12 estimates falling in the high or very high prevalence categories). Populations tested were often restricted to high-risk groups such as commercial sex workers and/or GUM attendees. Even studies reporting on similar subgroups of migrants found substantial differences in HIV prevalence. For example, UASC health check studies reported contrasting HIV prevalence levels (1.68%, 95%CI 0.46–5.92% [61] and 0.19%, 95%CI 0.05–0.68% [64], although the relatively small sample sizes mean that 95%CIs overlap). Prevalence among GUM attendees varied from 0.18% (95%CI 0.06–0.52% [60]) and 0.26% (95%CI 0.10–0.68%, female sex workers [52]) to 6.48% (95%CI 3.17–12.78%, GUM/Emergency Department attendee asylum seekers (64)) and 6.92% (95%CI 3.68–12.63%, male sex workers [53]). Less heterogeneity is observed by restricting to larger studies only (sample sizes more than 200) with HIV prevalence varying from 0.18% (95%CI 0.06–0.52%) [60] to 0.48% (95%CI 0.30–0.77%) [48].

HBV prevalence estimates again showed considerable variation, with study estimates between 0.00% (95%CI 0.00-9.64%) [65] and 8.93% (95%CI 5.85–13.39%) [66]. Estimates for larger studies (more than 1000 study participants) varied between 1.06% (95%CI 0.90–1.27%) [55] and 4.75% (95%CI 4.34–5.19%) [67]. Prevalence of HCV was lower, with studies reporting prevalence between 0.00% (four studies: [61, 65, 66, 68]) and 1.67% (95%CI 1.33–2.09%) [69]. Only two of the 11 included HCV estimates were above 0.50% [69, 70]. 

Table S4 summarises coinfection prevalence reported by studies estimating prevalence of multiple infections. Eight included studies provided estimates of prevalence of coinfections, or sufficient data from which to derive them [44, 48, 55, 64, 65, 68, 69, 71]. Excluding three studies with small sample sizes (*n* < 200 [64, 65, 71]), HBV-HCV coinfection prevalence varied between 0.00% [44, 48, 68] and 0.09% (95%CI 0.04–0.23%) [69] (*n* = 4). HIV-HBV coinfection prevalence was reported by two studies (0.02% [44] and 0.06% [48]). Two studies reported 0.00% coinfection prevalence of HCV (with active TB [44] and with IGRA, HIV and HBV [48]). IGRA-HIV and IGRA-HBV co-prevalence values were 0.14% and 0.80% respectively, reported by one study [48]. One study reported 0.00% coinfection of active TB with HIV, HBV and HCV (but based on only 9 diagnosed active TB cases [44]).

### Risk factor analysis: study characteristics

While Figs. 2 and 3a-b suggested no trend in infection prevalence/testing yield over time for IGRA, active TB, HIV and HBV, there was a general reduction in HCV prevalence reported over time by median year of data collection (Fig. 3c). This relationship was supported by the meta-regression analysis, which showed a significant decline in HCV prevalence over time for univariable and multivariable analyses (*p* < 0.0001 and 0 = 0.0009 respectively, Table 1). Prevalence estimates for active TB showed a borderline significant increase over time in multivariable (*p* = 0.03) but not univariable (*p* > 0.10) analysis. The meta-regression suggested no other significant changes in infection prevalence over time. Lower study quality was significantly associated with higher HIV prevalence (multivariable analysis, low quality vs. high: meta-regression coefficient 0.19, 95%CI 0.09–0.29, *p* = 0.0001; moderate vs. high: 0.12, 95%CI 0.02–0.21, *p* = 0.02). Of the 12 HIV estimates, three were of moderate and three were of low quality (Table S3, Supplementary Material). Moderate/low quality studies tended to have smaller sample sizes, but risk factors for HIV infection (e.g., sexual health clinic attendance) did not appear to be more common among the low/moderate quality studies. Study quality was not associated with prevalence for any other infection, except for active TB, where lower quality was borderline significantly associated with higher prevalence in multivariable analysis (low quality vs. high: meta-regression coefficient 0.09, 95%CI 0.01–0.17, *p* = 0.04; moderate vs. high: 0.08, 95%CI 0.02–0.14, *p* = 0.01). Given the small number of included studies, the influence of outlier studies of low/moderate quality [46, 61], and the second lowest prevalence estimate being from one extremely large, high quality study [45], are likely to be responsible for this relationship. There were no other significant associations except again for active TB, where studies testing pre-entry or at the time of UK entry reported lower prevalence than studies of post-entry testing (multivariable analysis, pre- vs. post-entry: meta-regression coefficient − 0.04, 95%CI -0.06 to -0.01, *p* = 0.01; at entry vs. post-entry: -0.07, 95%CI -0.11 to -0.03, *p* < 0.001). Pre-entry testing studies tended to use less sensitive diagnostic methods such as symptom-based and/or chest X-ray.

**Table 1:** Univariable and multivariable meta-regression analysis of factors potentially associated with prevalence of infection/test positivity. 95%CI – 95% confidence interval

	Univariable		Multivariable	
	Coefficient (95%CI)	*p* value	Coefficient (95%CI)	*p* value
**IGRA yield**				
Midpoint of study (year)	-0.01 (-0.02, 0.00)	0.05	-0.01 (-0.02, 0.01)	> 0.10
Study quality: low (vs. high)	0.05 (-0.10, 0.21)	> 0.10	0.13 (-0.02, 0.27)	0.092
Study quality: moderate (vs. high)	-0.01 (-0.14, 0.12)	> 0.10	-0.02 (-0.14, 0.11)	> 0.10
Pre-entry testing (vs. post UK arrival)	-	-	-	-
At entry testing (vs. post UK arrival)	-0.06 (-0.14, 0.01)	0.09	-0.06 (-0.17, 0.05)	> 0.10
**Active TB prevalence**				
Midpoint of study (year)	0.00 (0.00, 0.00)	> 0.10	**0.01 (0.00**,** 0.01)**	**0.03**
Study quality: low (vs. high)	0.01 (-0.05, 0.07)	> 0.10	**0.09 (0.01**,** 0.17)**	**0.04**
Study quality: moderate (vs. high)	0.01 (-0.06, 0.08)	> 0.10	**0.08 (0.02**,** 0.14)**	**0.01**
Pre-entry testing (vs. post UK arrival)	**-0.05 (-0.08**,** -0.02)**	**< 0.002**	**-0.04 (-0.06**,** -0.01)**	**0.01**
At entry testing (vs. post UK arrival)	**-0.04 (-0.07**,** -0.00)**	**0.03**	**-0.07 (-0.11**,** -0.03)**	**< 0.001**
**HIV prevalence**				
Midpoint of study (year)	0.00 (-0.01. 0.01)	> 0.10	0.00 (-0.01, 0.00)	> 0.10
Study quality: low (vs. high)	**0.18 (0.13**,** 0.23)**	**< 0.0001**	**0.19 (0.09**,** 0.29)**	**0.0001**
Study quality: moderate (vs. high)	**0.13 (0.07**,** 0.19)**	**< 0.0001**	**0.12 (0.02**,** 0.21)**	**0.02**
Pre-entry testing (vs. post UK arrival)	-0.06 (-0.26, 0.13)	> 0.10	0.00 (-0.10, 0.11)	> 0.10
At entry testing (vs. post UK arrival)	0.03 (-0.10, 0.16)	> 0.10	-0.02 (-0.11, 0.08)	> 0.10
**HBV prevalence**				
Midpoint of study (year)	0.00 (-0.01, 0.01)	> 0.10	0.00 (-0.01, 0.01)	> 0.10
Study quality: low (vs. high)	0.06 (-0.11, 0.23)	> 0.10	0.01 (-0.20, 0.23)	> 0.10
Study quality: moderate (vs. high)	-0.01 (-0.09, 0.08)	> 0.10	-0.01 (-0.11, 0.09)	> 0.10
Pre-entry testing (vs. post UK arrival)	0.00 (-0.15, 0.14)	> 0.10	-0.01 (-0.17, 0.16)	> 0.10
At entry testing (vs. post UK arrival)	0.05 (-0.04, 0.15)	> 0.10	0.05 (-0.08, 0.19)	> 0.10
**HCV prevalence**				
Midpoint of study (year)	**-0.01 (-0.01**,** 0.00)**	**< 0.0001**	**-0.01 (-0.01**,** 0.00)**	**0.0009**
Study quality: low (vs. high)	-0.03 (-0.15, 0.09)	> 0.10	0.01 (-0.10, 0.12)	> 0.10
Study quality: moderate (vs. high)	-0.05 (-0.12, 0.03)	> 0.10	-0.01 (-0.07, 0.05)	> 0.10
Pre-entry testing (vs. post UK arrival)	-0.01 (-0.09, 0.08)	> 0.10	0.00 (-0.04, 0.05)	> 0.10
At entry testing (vs. post UK arrival)	-0.03 (-0.11, 0.05)	> 0.10	0.00 (-0.06, 0.06)	> 0.10

### Risk factor analysis: prevalence estimates stratified by risk factors

Tables summarising infection prevalence estimates stratified by risk factors reported in included studies can be found in Supplementary Material Table S5 (IGRA positivity), Table S6 (active TB infection), Table S7 (HIV infection), Tables S8 and S9 (HBV infection) and Tables S10 and S11 (current and past HCV infection, respectively). There was considerable heterogeneity in risk factors presented. Prevalence was stratified by sex/gender and age in four IGRA [13, 48, 49, 58], one active TB [56], one HIV [48] and three HBV/HCV studies [48, 55, 69]. Prevalence stratified by region/country of birth/origin or nationality was reported by three IGRA [13, 58, 59], two active TB [44, 56], one HIV [44], five HBV [44, 59, 69, 72, 73] and two HCV studies [44, 69].

Other risk factors explored were TB incidence in country of origin [13, 58], BCG vaccination status [13, 58], contact with a TB case [56, 58], time since UK arrival [58, 69] and alcohol use [58, 68], while the following risk factors were reported by just a single study: ethnicity [55], travel to a TB-endemic country [58], employment status [58], smoking status [58], history of imprisonment [58], TB prevalence in country of screening [56] and migrant type [57]. Additionally, Kelly et al. reported prevalence amongst migrants according to the following risk factors for HBV infection: blood transfusion, surgery and dental work abroad, vaccination and hepatitis vaccination (which were not further defined in the article), past history of jaundice, family history of liver diseases, body/ear piercing and recreational/illicit substance use (Table S8 [68]).

Figure S2 (Supplementary Material) presents a heat map summarising prevalence of infection/test positivity for each infection, stratified by sex/gender (as reported by each original publication) and age. There were no obvious patterns of differences in infection prevalence by sex, other than one study finding higher active TB prevalence among women [56] and men having higher HBV prevalence [55, 69]. Trends in infection prevalence by age are hard to compare across studies because of the different age bands used by different studies. Despite IGRA testing being recommended for patients only up to 35 years in the UK [74], studies did report prevalence estimates for older age groups, with prevalence being higher than for younger groups [48, 49, 58]. Active TB prevalence increased with age [56].

For the three studies stratifying prevalence by age, HBV prevalence appeared slightly higher in the middle age groups (30 years up to around 60–65 years) [48, 55, 69]. No overall pattern was apparent for HCV.

Figure S3 (Supplementary Material) illustrates the relationship between study sample size and the number of risk factors by which prevalence was stratified for the included study population. It suggests that there is no relationship between sample size and number of risk factors for infection presented by studies, and that the majority of studies attempted no risk factor analysis for the study population included in our analysis.

**Fig. 4:**
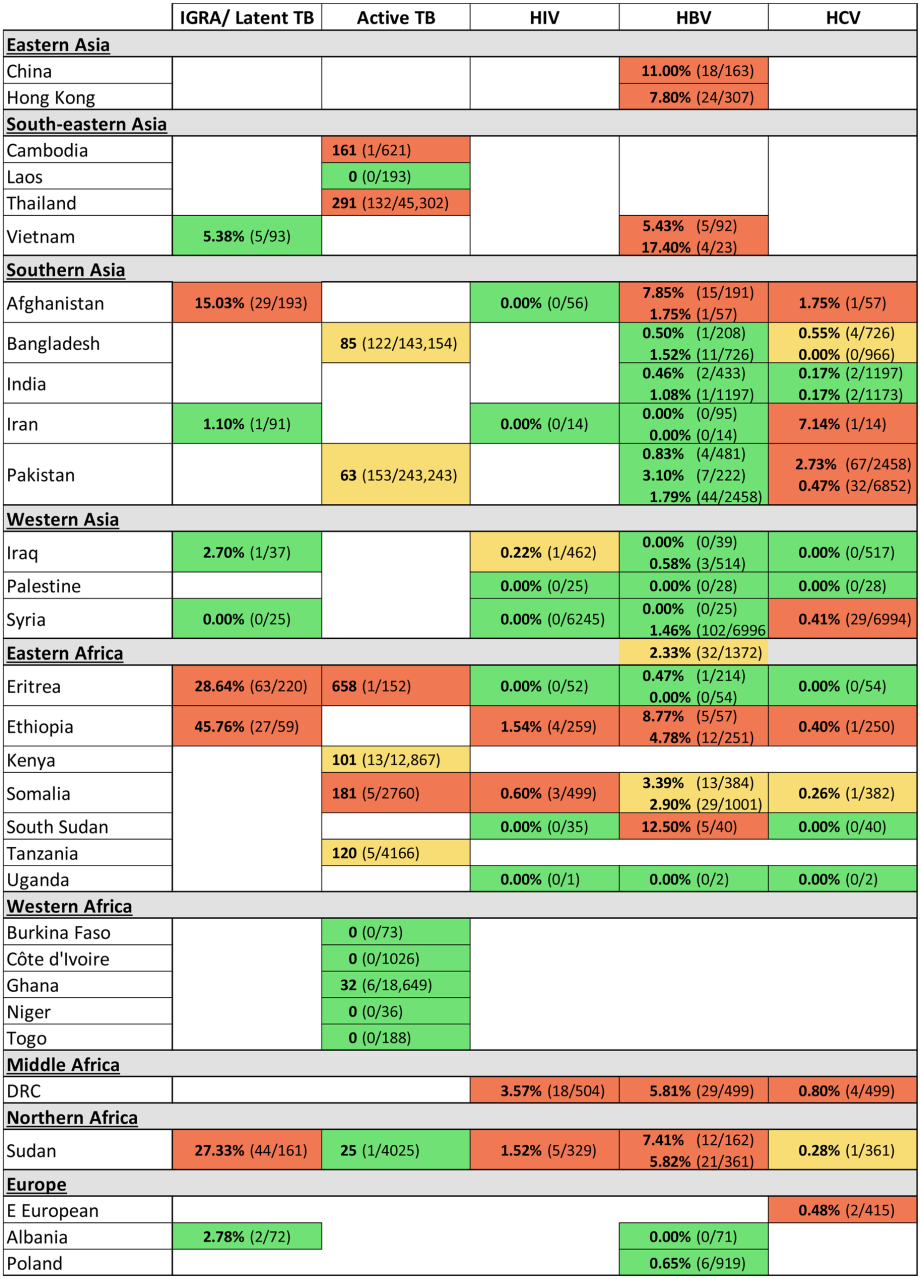
Heat map summarising risk of infection/test positivity for IGRA test yield, active TB, HIV, HBV and HCV infection, stratified by country of birth/origin reported by included studies. Prevalence is shown as % (number infected or testing positive over total screened) for IGRA test yield and HIV, HBV and HCV infection, and prevalence per 100,000 (number infected or testing positive over total screened) for active TB infection. Countries are arranged by United Nations geoscheme. Multiple estimates in one cell represent findings from independent studies. Further details of included studies [44, 56, 59, 69, 72, 73] are shown in Supplementary Material Tables S5-10, which also includes prevalence and test yield estimates for other risk factors. Colour scheme represents the categorisation described in the Methods section. HIV: red = extremely high prevalence (> 0.5%), orange = high (0.2–0.5%), green = low (< 0.2%) [34, 35]. HBV: red = high prevalence (≥ 5%), orange = intermediate (2-4.9%), green = low (< 2%) [36]. HCV: red = high prevalence (> 0.36%), orange = intermediate (> 0.18–0.36%), green = low (≤ 0.18%). IGRA: red = high yield (> 15%), orange = intermediate (> 7.5–15%), green = low (≤ 7.5%). Active TB: red = extremely high prevalence (≥ 150 per 100,000 population), orange = high (40–149 per 100,000), green = low (< 40 per 100,000) [40]. Multiple estimates within one cell have not been pooled because of their heterogeneity. However, simple pooling of estimates was conducted to determine colour categories for these cells

Figure 4 summarises prevalence of infection/test positivity stratified by country of birth/origin reported by studies, demonstrating that few studies attempted to stratify in this way. Fig. S4 (Supplementary Material) shows number of country of birth/origin-stratified prevalence estimates reported by included studies for the most common non-EU countries represented in the UK migrant population, alongside the infection burden in those countries of origin. Even for migrant groups from high-burden countries that make up a substantial share of UK migrants, country-specific prevalence estimates are often lacking. For example, no study reported Nigeria-specific infection prevalence estimates, despite Nigeria’s high infection burden and its position as the third most common non-EU country of origin among UK migrants. Care must be taken not to overinterpret Fig, 4 due to the small sample sizes of some of the strata. Nevertheless, it illustrates trends by region of birth/origin such as high levels of active TB and TB exposure among migrants from Eastern Africa and South-eastern Asia, and low prevalence in Western Africa. Southern Asian country of birth/origin estimates were generally high for HCV (three of five countries having high prevalence estimates), while Eastern, Middle and Northern African countries of birth/origin included many high prevalence levels for all infections.

Figure S5 (Supplementary Material) shows infection prevalence estimates for HIV, HBV and HCV, comparing prevalence among migrants with prevalence in their country of origin. The small samples for each country of origin stratum have resulted in wide 95%CI and uncertainty bounds for country prevalence are not shown, making interpretation difficult. However, it is notable that migrant prevalence estimates show no consistent pattern of being above or below prevalence in country of origin.

HIV prevalence estimates were higher for migrants from African regions than elsewhere but, as for TB/IGRA estimates, all strata were informed by a single study only [44]. For viral hepatitis, multiple studies presented several strata (two or three) for prevalence estimates. HBV and HCV estimates were heterogeneous between countries from the same region, particularly Southern Asia and Eastern Africa. HBV prevalence was higher among migrants from North, Middle and Eastern Africa (4.78–8.77% Ethiopia [44, 59], 12.50% South Sudan [44], 5.82–7.41% Sudan (44, 59), 5.81% DRC [44]) and Afghanistan (1.75–7.85% [44, 59]). Small sample sizes for some HCV strata restrict interpretation, but HCV prevalence was highest among migrants from Southern Asia, particularly Pakistan (0.47–2.73% [55, 69]) and Afghanistan (1.75% [44]).

## Discussion

We have systematically reviewed and summarised evidence on the prevalence of IGRA positivity and prevalence of active TB, HIV, HBV and HCV, in addition to risk factor-stratified prevalence estimates for migrants to the UK. Our review highlights the overall high levels of IGRA positivity and prevalence of all infections other than HCV, where prevalence amongst migrants is low and appears to be declining over time, consistent with observed decreases in chronic hepatitis C prevalence in the UK following expanded testing and access to curative direct-acting antiviral treatment, with chronic HCV prevalence in England having fallen by 57% between 2015 and 2023 [75]. IGRA positivity and HBV prevalence were particularly high. It also illustrates high levels of heterogeneity between studies, including a wide variety of migrant types who will have different patterns of risk, from UASC to specific subcategories of migrants, such as those attending sexual health services, and approaches to testing (including timing, eligibility and venue), all of which limit comparisons and generalisations. This could be explained by substantial variations in study populations and in methods of recruitment. While IGRA positivity estimates were relatively homogeneous, active TB prevalence showed great heterogeneity. For example, very low prevalence was reported for pre-entry migrant screening with diagnosis based on symptoms and chest X-ray [45], compared to UK-based testing, which may employ more sensitive diagnostic methods or a broader definition of active TB [48].

Although IGRA positivity was high in several studies, the corresponding rates of active TB were relatively low. This pattern is consistent with a substantial underlying burden of latent TB infection, with only a small proportion progressing to active disease. It is also important to note that IGRA is an imperfect test and is an indirect marker of *Mycobacterium tuberculosis* exposure rather than definitively indicating latent infection, so to maximise its positive predictive value, IGRA-based latent TB infection screening is reserved for those at sufficiently high risk of progressing to disease [76]. Uptake and completion of latent TB infection treatment among migrant populations may be limited by barriers such as access to care, fear of immigration consequences, language challenges, and competing social needs, although Baggaley et al. reported high acceptance of testing and treatment completion amongst migrants registering with primary care [48]. However, migrants less likely to register with a GP will be missed by such testing approaches and consequently, many individuals with latent TB infection remain untreated and at persistent risk of reactivation over time. Understanding these gaps in the latent TB infection care cascade is essential when interpreting IGRA prevalence and considering its implications for TB prevention strategies.

Studies are likely to have reported on a limited number of risk factors because of restricted sample sizes. However, the choice of risk factors varied widely and there were inconsistent category boundaries (for example, age group cut-offs), making comparisons difficult.

There were also gaps in terms of reporting infection prevalence for migrants from some countries with high numbers living in the UK, and which have high infection prevalence in their country of origin. We would have expected to see reporting of more risk factors with increasing study sample size, but that did not appear to be the case. The risk factor analysis revealed that very few studies explored differences in prevalence beyond basic demographic factors (age, sex, ethnicity, country of origin/birth), such as reporting of risk behaviours (e.g., for BBVs, injecting drug use, various sexual risk behaviours). This is likely due to limitations in data collection, particularly for studies analysing routine data. Reporting prevalence stratified by additional risk factors would help identify which migrant groups are at greatest risk of infection, informing targeted interventions such as raising testing awareness among higher-risk groups.

The prevalence estimates we have compiled in this review each offer a snapshot of prevalence amongst migrants. However, migrants are a highly heterogeneous, dynamic population; countries of origin of UK-bound migrants change over time, and prevalence of infection in these countries may also change. Over the past two decades, the demographic profile of migrants to the UK has shifted substantially. Following EU enlargement in 2004, migration was dominated by arrivals from Central and Eastern Europe (e.g., Poland, Romania, Lithuania), where the prevalence of TB, hepatitis B, hepatitis C, and HIV is comparatively low. However, from the mid-2010s onwards, migration increasingly originated from South Asia (particularly India, Pakistan and Bangladesh), sub-Saharan Africa (notably Nigeria, Ghana, Somalia, and Eritrea) and the Middle East. These regions have considerably higher burdens of TB and viral hepatitis, and in some settings higher HIV prevalence as well. As a result, prevalence estimates reported in studies spanning 2004–2025 may partly reflect these changing migrant origins rather than genuine temporal changes in infection risk. The considerable heterogeneity between studies may have masked any changes in prevalence over time, with the exception of decreasing HCV prevalence.

These dynamics mean there is a need for continual monitoring of key infections such as TB and BBVs among this and other higher risk populations, as circumstances potentially change. In addition, surveillance should encompass the prevalence of other infections that disproportionately affect migrant populations, including helminthiases, together with the proportion of infections that exhibit drug resistance, to inform appropriate public health responses. More generally, the disruption to health services arising from drastic and abrupt cuts in global health funding threatens to reverse progress with infectious disease prevention programmes [77, 78], so increases in prevalence are possible. Furthermore, an HCV modelling analysis among another BBV high-risk population (people who inject drugs) has shown the importance of maintaining test and treat provision as we approach the elimination threshold, to prevent a U-turn in prevalence [79].

A multifaceted approach, providing multiple opportunities to test, is likely to be more successful than testing and treating these infections in silos, and has been shown to be acceptable to migrant populations when registering with primary care [48, 80]. The COMBAT-ID study reported comparatively little coinfection (0.06% tested HIV-HBV co-positive, 0.14% HIV-IGRA co-positive and 0.80% HBV-IGRA co-positive, with no coinfections involving HCV). BBV prevalence was substantial amongst those IGRA-negative and actually higher for all three BBVs for those not IGRA tested compared to those tested (IGRA testing is recommended only for patients up to 35 years in the UK [74]). HIV, HBV and HCV prevalence were 0.70%, 3.99% and 0.00% among IGRA-positive patients respectively, compared to 0.12%, 1.94% and 0.06% among IGRA-negative patients and 0.85%, 4.86% and 0.37% amongst patients not IGRA tested [48]. This suggests that a multiple infection rather than sequential approach to IGRA and BBV infection testing is appropriate, so BBV infections are not missed.

There are some limitations to our analysis. We may have missed unpublished studies and many prevalence estimates stratified by risk factors could be derived by re-analysing existing datasets, but that was beyond the scope of the current analysis. We included prevalence estimates from up to 21 years ago, but older studies may not reflect the situation now, given the dynamic, heterogeneous composition of migrants to the UK and changes in prevalence of infection and risk factors for infection acquisition in migrants’ countries of origin and along migration routes to the UK. Similarly, although we could have included infection prevalence estimates for migrants to other high-income countries, substantial differences in migrant population composition, such as variation in countries of origin, even among Western European destinations, mean that prevalence estimates from one country cannot be generalised to others without verification that the migrant populations are comparable. 

Given the small number of studies available for certain outcomes, the meta-regression findings should be considered hypothesis-generating rather than confirmatory. As meta-regression was conducted using study-level data, observed associations represent ecological relationships and should not be interpreted as individual-level or causal effects. The categorisation of IGRA positivity and HCV prevalence into qualitative descriptors (e.g., low, intermediate and high HCV prevalence levels) was author-defined for descriptive purposes in the absence of universally accepted thresholds, and these categories should not be interpreted as validated clinical or policy cut-offs. Our risk factor analysis was limited by small sample sizes in many cases; many papers reporting relevant data had different research aims, meaning data were stratified in a different way which could not fulfil our inclusion criteria. Further research may include revisiting these datasets to analyse risk factors using standardised categories, data access permitting.

## Conclusions

While prevalence estimates for each infection showed high heterogeneity, to be expected given the wide variation in study designs and study populations, prevalence of these infections, particularly IGRA yield and HBV, is high. This underscores the need to maintain effective monitoring, testing and treatment for key infections among migrant populations. Further work is needed to determine which testing strategies are most cost-effective, acceptable to migrants, and appropriately targeted by risk of infection, particularly in the context of a rapidly changing epidemiological and demographic landscape. To generate this evidence, more detailed reporting of infection prevalence by migrant subgroup and relevant risk factors is needed.

## Supplementary Information

Below is the link to the electronic supplementary material.

Supplementary Material 1

## Data Availability

All data generated or analysed during this study are included in this published article and its supplementary information files.

## Abbreviations

95%CI: 95% confidence interval
BBV: Blood-borne virus
DBS: Neonatal dried blood spot testing
E: East
ED: Emergency Department
ESOL: English for Speaker of Other Languages course
exc: excluding
FSW: Female sex worker
GP: General practice/primary care
GUM: Genitourinary medicine or sexual health clinic
HBV: Hepatitis B virus
HCV: hepatitis C virus
HIV: Human immunodeficiency virus
HPU: Health Protection Unit
IAC: Initial Accommodation Centre for UK asylum seekers
IGRA: Interferon gamma release assay
IOM: International Organization for Migration
IQR: Interquartile range
MeSH: Medical Subject Heading
MSW: Male sex worker
NICE: National Institute for Health and Care Excellence
NS: Not stated
PRISMA: Preferred Reporting Items for Systematic Reviews and Meta-Analyses
SSA: Sub Saharan Africa
TB: Tuberculosis
UASC: Unaccompanied asylum seeking children
UKHSA: United Kingdom Health Security Agency
y: years

## References

[1] Nazareth J, Baggaley RF, Divall P, Pan D, Martin CA, Volik M, et al. What is the evidence on existing national policies and guidelines for delivering effective tuberculosis, HIV and viral hepatitis services for refugees and migrants among member States of the WHO European Region? WHO Health Evidence Network Synthesis Reports. Copenhagen. 2021. 35263069

[2] ECDC. European Centre for Disease Prevention and Control (ECDC). Public health guidance on HIV, hepatitis B and C testing in the EU/EEA: an integrated approach. Available from: https://www.ecdc.europa.eu/en/publications-data/public-health-guidance-hiv-hepatitis-b-and-c-testing-eueea Accessed 26 October 2025. 2018.

[3] Simões D, Raben D, Moran AB, Imaz A, Stengaard AR, Raahauge A, et al. The HepHIV 2023 Madrid conference: a call to action for political leadership in reaching the sustainable development goals on earlier testing and linkage to care for HIV, viral hepatitis, and sexually transmitted infections. HIV Med. 2024;25(10):1169-76. 10.1111/hiv.13683 38923107

[4] UKHSA. United Kingdom Health Security Agencey (UKHSA). Research and analysis report: 1. Tuberculosis incidence and epidemiology, England. 2024. Available from: https://www.gov.uk/government/publications/tuberculosis-in-england-2025-report/1-tuberculosis-incidence-and-epidemiology-england-2024 Accessed 14 December 2025.

[5] Santoso D, Asfia S, Mello MB, Baggaley RC, Johnson CC, Chow EPF, et al. HIV prevalence ratio of international migrants compared to their native-born counterparts: A systematic review and meta-analysis. EClinicalMedicine. 2022;53:101661. 36147629 10.1016/j.eclinm.2022.101661 PMC9486043

[6] UKHSA. United Kingdom Health Security Agency (UKHSA). Research and analysis: Hepatitis B in England 2024. 2024. Available from: https://www.gov.uk/government/publications/hepatitis-b-in-england/hepatitis-b-in-england-2024?utm_source=chatgpt.com Accessed 14 December 2025. 2024.

[7] Cortina-Borja M, Williams D, Peckham CS, Bailey H, Thorne C. Hepatitis C virus seroprevalence in pregnant women delivering live-born infants in North Thames, England in 2012. Epidemiol Infect. 2016;144(3):627–34. 26178148 10.1017/S0950268815001557 PMC4714297

[8] WHO. World Health Organization. Report on the health of refugees and migrants in the WHO European Region: No public health without refugee and migrant health. 2018. Available at: https://iris.who.int/handle/10665/311347 Accessed 6 September 2024.

[9] Delpech V, Brown AE, Croxford S, Chau C, Polavarapu V, Cooper N, et al. Quality of HIV care in the United Kingdom: key indicators for the first 12 months from HIV diagnosis. HIV Med. 2013;14(Suppl 3):19–24. 24033898 10.1111/hiv.12070

[10] Kruijshaar ME, Abubakar I. Increase in extrapulmonary tuberculosis in England and Wales 1999–2006. Thorax. 2009;64(12):1090–5. 19850965 10.1136/thx.2009.118133

[11] NICE. National Institute for Health and Care Excellence. Hepatitis B and C testing: people at risk of infection. Public health guideline [PH43]. Published December 2012, last updated March 2013. 2012. Available from: https://www.nice.org.uk/guidance/ph43/chapter/Recommendations#introduction%20reference%20added%20to%20the%20converted%20endnote%20lib,%20not%20letting%20me%20add%20from%20home Accessed 9 September 2024.

[12] National Collaborating Centre for Chronic Conditions. Tuberculosis: Clinical Diagnosis and Management of Tuberculosis, and Measures for Its Prevention and Control. London: Royal College of Physicians. NICE Clinical Guidelines, No. 117. 2011. Available from: https://www.ncbi.nlm.nih.gov/books/NBK97852/ Accessed 9 September 2024.

[13] Pareek M, Watson JP, Ormerod LP, Kon OM, Woltmann G, White PJ, et al. Screening of immigrants in the UK for imported latent tuberculosis: a multicentre cohort study and cost-effectiveness analysis. Lancet Infect Dis. 2011;11(6):435–44. 21514236 10.1016/S1473-3099(11)70069-X PMC3108102

[14] NICE, Tuberculosis. NICE Guideline No. 33. ISBN-13: 978-1-4731-5741-5. Published January 2016, last updated February 2024. 2016. Available from: https://www.ncbi.nlm.nih.gov/books/NBK553007/ Accessed 6 September 2024.

[15] UKHSA. UK Health Security Agency. Policy paper: Tuberculosis (TB): action plan for England, 2021 to 2026. 2023. Available from: https://www.gov.uk/government/publications/tuberculosis-tb-action-plan-for-england/tuberculosis-tb-action-plan-for-england-2021-to-2026#priority-2-prevent-tb Accessed 9 September 2024.

[16] NICE. National Institute for Health and Care Excellence. HIV testing: increasing uptake among people who may have undiagnosed HIV. NICE guideline [NG60]. 2016. Available from: https://www.nice.org.uk/guidance/ng60 Accessed 9 September 2024. 41428834

[17] BHIVA, British HIV, Association. BHIVA/BASHH/BIA Adult HIV Testing guidelines 2020. 2020. Available from: https://www.bhiva.org/HIV-testing-guidelines Accessed 9 September 2024.

[18] PHE. Public Health England Guidance. HIV: testing. Published. January 2014, updated November 2017. 2014. Available from: https://www.gov.uk/guidance/hiv-testing#recommendations Accessed 9 September 2024.

[19] UKHSA. UK Health Security Agency, Hepatitis B. September in England – 2023 report. Working to eliminate hepatitis B as a public health threat. Data to end of 2021.2023. Available from: https://assets.publishing.service.gov.uk/media/63dba059d3bf7f0704f31d37/Hepatitis_B_in_England_2023.pdf Accessed 9 2024.

[20] Advisory Group on Hepatitis. Case-finding for hepatitis B and C virus infection in minority ethnic populations in the United Kingdom. London: Advisory Group on Hepatitis; 2009.

[21] Ford C, Halliday K, Foster G, Gore C, Jack K, Rowan N, et al. Royal College of General Practitioners. Guidance for the prevention, testing, treatment and management of hepatitis C in primary care. 2007. Available from the Health Research Board National Drugs Library at: https://www.drugsandalcohol.ie/13637/ Accessed 9 September 2024.

[22] UKHSA. UK Health Security Agency Official Statistics. National quarterly report of tuberculosis in England: Quarter 3, 2023 provisional data. Updated 25 July 2024. 2023. Available from: https://www.gov.uk/government/statistics/tuberculosis-in-england-national-quarterly-reports/national-quarterly-report-of-tuberculosis-in-england-quarter-3-2023-provisional-data#:~:text=Similar%20patterns%20have%20been%20observed,East%20Midlands%20(10.9%25%20decrease) Accessed 9 September 2024.

[23] UK Department of Health and Social Care. Policy Paper. Towards Zero: the HIV Action Plan for England – 2022 to 2025. Published 1 December 2021. 2021. Available from: https://www.gov.uk/government/publications/towards-zero-the-hiv-action-plan-for-england-2022-to-2025 Accessed 26 November 2024.

[24] UKHSA. UK Health Security Agency Research and analysis. Hepatitis C in England 2023: working to eliminate hepatitis C as a public health problem. Data to end of 2022. London, UKHSA. 2024. Available from: https://www.gov.uk/government/publications/hepatitis-c-in-the-uk/hepatitis-c-in-england-2023#:~:text=multi%2Dstakeholder%20group.-,Introduction,before%201986%20in%20the%20UK Accessed 9 September 2024.

[25] Lonnroth K, Migliori GB, Abubakar I, D’Ambrosio L, de Vries G, Diel R, et al. Towards tuberculosis elimination: an action framework for low-incidence countries. Eur Respir J. 2015;45(4):928–52. 25792630 10.1183/09031936.00214014 PMC4391660

[26] Lonnroth K, Mor Z, Erkens C, Bruchfeld J, van der Nathavitharana RR, et al. Tuberculosis in migrants in low-incidence countries: epidemiology and intervention entry points. Int J Tuberc Lung Dis. 2017;21(6):624–37. 28482956 10.5588/ijtld.16.0845

[27] Office for Health Improvement and Disparities, Guidance. Tuberculosis (TB): migrant health guide. Published July 2014, last updated June 2023. 2023. Available from: https://www.gov.uk/guidance/tuberculosis-tb-migrant-health-guide#:~:text=LTBI%2520testing%2520and%2520treatment%26text=There%2520is%2520evidence%2520that%2520LTBI,for%2520LTBI%2520in%2520the%2520UK Accessed 9 September 2024.

[28] UKHSA. UK Health Security Agency Official Statistics. National quarterly report of tuberculosis in England: quarter 2, 2024, provisional data. Updated 25 July 2024. 2024. Available from: https://www.gov.uk/government/statistics/tuberculosis-in-england-national-quarterly-reports/national-quarterly-report-of-tuberculosis-in-england-quarter-2-2024-provisional-data Accessed 9 September 2024.

[29] Page MJ, McKenzie JE, Bossuyt PM, Boutron I, Hoffmann TC, Mulrow CD, et al. The PRISMA 2020 statement: an updated guideline for reporting systematic reviews. BMJ. 2021;372:n71. 33782057 10.1136/bmj.n71 PMC8005924

[30] IOM. International Organization for Migration (IOM). International Migration Law No. 34 - Glossary on Migration. ISSN 1813–2278. 2019. Available from: https://publications.iom.int/books/international-migration-law-ndeg34-glossary-migration Accessed 11 July 2025.

[31] Ouzzani M, Hammady H, Fedorowicz Z, Elmagarmid A. Rayyan - a web and mobile app for systematic reviews. Syst Reviews. 2016;5:210. 10.1186/s13643-016-0384-4 PMC5139140 27919275

[32] Joanna Briggs Institute Critical Appraisal Tools for Prevalence Studies. 2020. Available from: https://jbi.global/critical-appraisal-tools Accessed 22 July 2024.

[33] Munn Z, Moola S, Lisy K, Riitano D, Tufanaru C. Joanna Briggs Institute. Chapter 5: Systematic reviews of prevalence and incidence. In: Aromataris E, Munn Z, editors. JBI Manual for Evidence Synthesis. 2020.

[34] NICE. National Institute for Health and Care Excellence (NICE) and Public Health England (PHE). HIV testing: increasing uptake among people who may have undiagnosed HIV. NICE guideline NG60. Published 1 December 2016. 2016. Available from: www.nice.org.uk/guidance/ng60 Accessed 25 January 2026. 41428834

[35] BHIVA, British HIV. Association (BHIVA)/British Association for Sexual Health and HIV (BASHH)/British Infection Association (BIA). Adult HIV Testing Guidelines 2020. 2020. Available from: https://bhiva.org/clinical-guideline/hiv-testing-guidelines/ Accessed 25 January 2026.

[36] WHO. World Health Organization (WHO). WHO Guidelines on Hepatitis B and C Testing. Geneva: World Health Organization. Table 1: Summary of recommendations on testing for chronic hepatitis B and C virus infection. 2017. Available from: https://www.who.int/publications/i/item/9789241549981 Accessed 24 January 2026.

[37] Mitchell H, Costella A, Clancy I, Harris R, Hibbert M, Powell A, et al. Hepatitis C in England 2024: working to eliminate hepatitis C as a public health problem. Data to end of 2023. London: UK Health Security Agency (UKHSA). 2025. Available from: https://www.gov.uk/government/publications/hepatitis-c-in-the-uk/hepatitis-c-in-england-2024#acknowledgments Accessed 25 January 2026.

[38] Hinks TS, Varsani N, Godsiff DT, Bull TC, Nash KL, McLuckie L, et al. High background rates of positive tuberculosis-specific interferon-gamma release assays in a low prevalence region of UK: a surveillance study. BMC Infect Dis. 2012;12:339. 23216965 10.1186/1471-2334-12-339 PMC3537536

[39] Thomas A, Halliday A, Clapp G, Symonds R, Hopewell-Kelly N, McGrath C, et al. High Mycobacterium bovis Exposure but Low IGRA Positivity in UK Farm Workers. Zoonoses Public Health. 2025;72(4):369–78. 40007044 10.1111/zph.13214 PMC12016005

[40] UKHSA. UK Health Security Agency (UKHSA). Tuberculosis (TB): migrant health guide. 2014. Available from: https://www.gov.uk/guidance/tuberculosis-tb-migrant-health-guide Accessed 25 January 2026.

[41] ONS. Office for National Statistics (ONS). Counties and Unitary Authorities. (May 2023) Boundaries UK BGC. 2023. Available from: https://www.data.gov.uk/dataset/85228aec-fe0e-49bf-9455-df000d61e731/counties-and-unitary-authorities-may-2023-boundaries-uk-bgc Accessed 27 November 2025.

[42] UNAIDS. UNAIDS estimates, Joint United Nations Programme on HIV/AIDS (UNAIDS.), uri: aidsinfo.unaids.org, publisher: UNAIDS, data accessed via The World Bank. Available from: https://data.worldbank.org/indicator/SH.DYN.AIDS.ZS Date accessed: 27 January 2026.

[43] Coalition for Global Hepatitis Elimination and The Task Force For Global Health. Global Statistics. Available from: https://www.globalhep.org/data-profiles Accessed 27 January 2026.

[44] Crawshaw AF, Pareek M, Were J, Schillinger S, Gorbacheva O, Wickramage KP, et al. Infectious disease testing of UK-bound refugees: a population-based, cross-sectional study. BMC Med. 2018;16(1):143. 30149810 10.1186/s12916-018-1125-4 PMC6112114

[45] Menezes D, Zenner D, Aldridge R, Anderson SR, de Vries G, Erkens C, et al. Country differences and determinants of yield in programmatic migrant TB screening in four European countries. Int J Tuberc Lung Dis. 2022;26(10):942–8. 36163670 10.5588/ijtld.22.0186 PMC7615138

[46] Severi E, Maguire H, Ihekweazu C, Bickler G, Abubakar I. Outcomes analysis of new entrant screening for active tuberculosis in Heathrow and Gatwick airports, United Kingdom 2009/2010. BMC Infect Dis. 2016;16:178. 27102741 10.1186/s12879-016-1506-2 PMC4840491

[47] Martyn E, O’Regan S, Harris P, Leonard M, Veitch M, Sultan B, et al. Hepatitis B virus (HBV) screening, linkage and retention-in-care in inclusion health populations: Evaluation of an outreach screening programme in London. J Infect. 2024;88(2):167–72. 38159579 10.1016/j.jinf.2023.12.012 PMC7615690

[48] Baggaley RF, Martin CA, Eborall HC, Gohar M, Aziz K, Fahad M, et al. Community-based testing of migrants for infectious diseases (COMBAT-ID): observational cohort study measuring the effectiveness of routine testing for infectious diseases among migrants attending primary care. eClinicalMedicine. 2025;Online first103253 May 30, 2025. 10.1016/j.eclinm.2025.103253 PMC12273839 40687739

[49] Barry SM, Davies G, Barry TD, Evans J, Backx M, Brouns M, et al. Outcomes from a national screening program for Ukrainian refugees at risk of drug resistant tuberculosis in Wales. Thorax. 2023;79(1):86–9. 37344177 10.1136/thorax-2023-220161 PMC10804008

[50] Berrocal-Almanza LC, Harris RJ, Collin SM, Muzyamba MC, Conroy OD, Mirza A, et al. Effectiveness of nationwide programmatic testing and treatment for latent tuberculosis infection in migrants in England: a retrospective, population-based cohort study. Lancet Public Health. 2022;7(4):e305–15. 35338849 10.1016/S2468-2667(22)00031-7 PMC8967722

[51] O’Shea MK, Fletcher TE, Beeching NJ, Dedicoat M, Spence D, McShane H, et al. Tuberculin skin testing and treatment modulates interferon-gamma release assay results for latent tuberculosis in migrants. PLoS ONE. 2014;9(5):e97366. 24816576 10.1371/journal.pone.0097366 PMC4016319

[52] Mc Grath-Lone L, Marsh K, Hughes G, Ward H. The sexual health of female sex workers compared with other women in England: analysis of cross-sectional data from genitourinary medicine clinics. Sex Transm Infect. 2014;90(4):344–50. 24493858 10.1136/sextrans-2013-051381 PMC4033115

[53] Mc Grath-Lone L, Marsh K, Hughes G, Ward H. The sexual health of male sex workers in England: analysis of cross-sectional data from genitourinary medicine clinics. Sex Transm Infect. 2014;90(1):38–40. 24273126 10.1136/sextrans-2013-051320 PMC3913221

[54] Berrocal-Almanza LC, Harris R, Lalor MK, Muzyamba MC, Were J, O’Connell AM, et al. Effectiveness of pre-entry active tuberculosis and post-entry latent tuberculosis screening in new entrants to the UK: a retrospective, population-based cohort study. Lancet Infect Dis. 2019;19(11):1191–201. 31471131 10.1016/S1473-3099(19)30260-9

[55] Flanagan S, Kunkel J, Appleby V, Eldridge SE, Ismail S, Moreea S, et al. Case finding and therapy for chronic viral hepatitis in primary care (HepFREE): a cluster-randomised controlled trial. Lancet Gastroenterol Hepatol. 2019;4(1):32–44. 30477810 10.1016/S2468-1253(18)30318-2

[56] Aldridge RW, Zenner D, White PJ, Muzyamba MC, Loutet M, Dhavan P, et al. Prevalence of and risk factors for active tuberculosis in migrants screened before entry to the UK: a population-based cross-sectional study. Lancet Infect Dis. 2016;16(8):962–70. 27013215 10.1016/S1473-3099(16)00072-4

[57] Zenner D, Brals D, Nederby-Ohd J, Menezes D, Aldridge R, Anderson SR, et al. Drivers determining tuberculosis disease screening yield in four European screening programmes: a comparative analysis. Eur Respir J. 2023;62(4). 10.1183/13993003.02396-2022 PMC10568038 37230498

[58] Pareek M, Bond M, Shorey J, Seneviratne S, Guy M, White P, et al. Community-based evaluation of immigrant tuberculosis screening using interferon gamma release assays and tuberculin skin testing: observational study and economic analysis. Thorax. 2013;68(3):230–9. 22693179 10.1136/thoraxjnl-2011-201542 PMC5741173

[59] Eisen S, Williams B, Cohen J. Infections in Asymptomatic Unaccompanied Asylum-seeking Children in London 2016–2022. Pediatr Infect Dis J. 2023;42(12):1051–5. 37725799 10.1097/INF.0000000000004087

[60] Burns FM, Mercer CH, Evans AR, Gerry CJ, Mole R, Hart GJ. Increased attendances of people of eastern European origin at sexual health services in London. Sex Transm Infect. 2009;85(1):75–8. 18768538 10.1136/sti.2007.029546

[61] Pinto AC, Seery P, Foster C. Evaluating a clinic for unaccompanied asylum seeking children (UASC): the importance of infectious disease screening and a holistic approach to care. Abstract 769. Arch Dis Child. 2022;107:A327.

[62] Usdin M, Dedicoat M, Gajraj R, Harrison P, Kaur H, Duffield K, et al. Latent tuberculous screening of recent migrants attending language classes: a cohort study and cost analysis. Int J Tuberc Lung Dis. 2017;21(2):175–80. 28234081 10.5588/ijtld.16.0398

[63] WHO. World Health Organization. Global tuberculosis report 2025. 2025. Available from: https://www.who.int/teams/global-programme-on-tuberculosis-and-lung-health/data#:~:text=Data%20reported%20by%20countries%20to,Global%20tuberculosis%20report%20was%20written. Accessed 14 December 2025.

[64] Amrose A, Ramshaw S, Dwyer E, Bull L. Prevalence and presentations of HIV in asylum seekers in the London borough of Hounslow. HIV Med. 2023;24:78. Abstract P089. British HIV Association (BHIVA) Spring Conference 2023.

[65] Hargreaves S, Seedat F, Car J, Escombe R, Hasan S, Eliahoo J, et al. Screening for latent TB, HIV, and hepatitis B/C in new migrants in a high prevalence area of London, UK: a cross-sectional study. BMC Infect Dis. 2014;14:657. 25466442 10.1186/s12879-014-0657-2 PMC4261901

[66] Vedio AB, Ellam H, Rayner F, Stone B, Kudesia G, McKendrick MW, et al. Hepatitis B: report of prevalence and access to healthcare among Chinese residents in Sheffield UK. J Infect Public Health. 2013;6(6):448–55. 23999342 10.1016/j.jiph.2013.05.004

[67] Evlampidou I, Hickman M, Irish C, Young N, Oliver I, Gillett S, et al. Low hepatitis B testing among migrants: a cross-sectional study in a UK city. Br J Gen Pract. 2016;66(647):e382–91. 27025556 10.3399/bjgp16X684817 PMC4871303

[68] Kelly C, Mathew S, Petrova M, Shafi S, Nicholls M, Dar O, et al. Exploring awareness and prevalence of chronic viral hepatitis in a UK based Nepali population - lessons learned for future models in engaging migrant communities. Clin Med (Lond). 2023;23(6):563–70. 38065610 10.7861/clinmed.2022-0356 PMC11046676

[69] Uddin G, Shoeb D, Solaiman S, Marley R, Gore C, Ramsay M, et al. Prevalence of chronic viral hepatitis in people of south Asian ethnicity living in England: the prevalence cannot necessarily be predicted from the prevalence in the country of origin. J Viral Hepat. 2010;17(5):327–35. 20002307 10.1111/j.1365-2893.2009.01240.x

[70] O’Leary MC, Sarwar M, Hutchinson SJ, Weir A, Schofield J, McLeod A, et al. The prevalence of hepatitis C virus among people of South Asian origin in Glasgow - results from a community based survey and laboratory surveillance. Travel Med Infect Dis. 2013;11(5):301–9. 24007935 10.1016/j.tmaid.2013.08.001

[71] Hargreaves S, Nellums LB, Johnson C, Goldberg J, Pantelidis P, Rahman A, et al. Delivering multi-disease screening to migrants for latent TB and blood-borne viruses in an emergency department setting: A feasibility study. Travel Med Infect Dis. 2020;36:101611. 32126293 10.1016/j.tmaid.2020.101611 PMC7493708

[72] Cochrane A, Evlampidou I, Irish C, Ingle SM, Hickman M. Hepatitis B infection prevalence by country of birth in migrant populations in a large UK city. J Clin Virol. 2015;68:79–82. 26071342 10.1016/j.jcv.2015.05.009

[73] McPherson S, Valappil M, Moses SE, Eltringham G, Miller C, Baxter K, et al. Targeted case finding for hepatitis B using dry blood spot testing in the British-Chinese and South Asian populations of the North-East of England. J Viral Hepat. 2013;20(9):638–44. 23910648 10.1111/jvh.12084

[74] PHE. Public Health England. Latent TB testing and treatment for migrants: a practical guide for commissioners and practitioners. 2015. Available from: https://assets.publishing.service.gov.uk/government/uploads/system/uploads/attachment_data/file/442192/030615_LTBI_testing_and_treatment_for_migrants_1.pdf Accessed 7 March 2025.

[75] UKHSA. UK Health Security Agency (UKHSA). Hepatitis C in England 2024. Updated 12 March 2025. 2025. Available from: https://www.gov.uk/government/publications/hepatitis-c-in-the-uk/hepatitis-c-in-england-2024 Accessed 12 December 2025.

[76] Pai M, Denkinger CM, Kik SV, Rangaka MX, Zwerling A, Oxlade O, et al. Gamma interferon release assays for detection of Mycobacterium tuberculosis infection. Clin Microbiol Rev. 2014;27(1):3–20. 24396134 10.1128/CMR.00034-13 PMC3910908

[77] McClure M, Gandhi M. Threat of HIV and tuberculosis drug resistance after US funding cuts. Lancet Infect Dis. 2025;25(5):e256–7. 40122094 10.1016/S1473-3099(25)00209-9

[78] Zumla A, Sahu S, Yeboah-Manu D, Goletti D, Nyasulu PS, Mfinanga S, et al. Breaking dependency: strengthening the global tuberculosis response in the face of USAID cuts. Lancet. 2025;405(10483):958–61. 40023179 10.1016/S0140-6736(25)00335-6

[79] Cousien A, Tran VC, Deuffic-Burban S, Jauffret-Roustide M, Dhersin JS, Yazdanpanah Y. Hepatitis C treatment as prevention of viral transmission and liver-related morbidity in persons who inject drugs. Hepatology. 2016;63(4):1090–101. 26390137 10.1002/hep.28227

[80] Eborall H, Wobi F, Ellis K, Willars J, Abubakar I, Griffiths C, et al. Integrated screening of migrants for multiple infectious diseases: qualitative study of a city-wide programme. eclinicalmedicine. 2020;21:100315. 32322806 10.1016/j.eclinm.2020.100315 PMC7170938

[81] NHS England website. National latent tuberculosis infection testing and treatment programme. Available from: https://www.england.nhs.uk/ourwork/prevention/tuberculosis-programme/national-latent-tuberculosis-infection-testing-and-treatment-programme/. Accessed 23 February 2025.

