# Supplementary material for: Prevalence and risk factors for key infectious diseases amongst migrants to the UK: a systematic review
Source: BMC Infect Dis. 2026 Mar 3;26:737. doi: 10.1186/s12879-026-12953-z (PMC13063883; doi:10.1186/s12879-026-12953-z)
Supplement: Supplementary file 1 — Supplementary Material 1 [file 12879_2026_12953_MOESM1_ESM.docx]

**Supplementary Material**

**Prevalence and risk factors for key infectious diseases amongst migrants to the UK: a systematic review**

Baggaley RF^a,b,c^, Hooper CM^d^, Silva L^b,c^, Lal Z^b,c^, Bird P^e^, Menezes D^a^, Zenner D^f^, Martin CA^b,c,g,h,i^, Pareek M^b,c,g,h,i^

1. Institute of Health Informatics, University College London, London, UK
2. Division of Public Health and Epidemiology, School of Medical Sciences, University of Leicester, Leicester, UK
3. Development Centre for Population Health, University of Leicester, Leicester, UK
4. Infection and Immunity Research Unit, St George’s University of London, London, UK
5. Department of Clinical Microbiology, University Hospitals of Leicester NHS Trust, Leicester, UK
6. Wolfson Institute of Population Health, Queen Mary University of London, London, UK
7. Department of Infection and HIV Medicine, University Hospitals of Leicester NHS Trust, Leicester, UK
8. NIHR Applied Research Collaboration East Midlands, University of Leicester, Leicester, UK
9. NIHR Leicester Biomedical Research Centre (BRC), University of Leicester, Leicester, UK

**Figure S1** Geographical distribution of included study settings across the UK.

**Figure S1 alt text** Choropleth map of the United Kingdom showing the geographical distribution of included study settings by local area. Areas are shaded from very light to dark blue to indicate increasing numbers of studies (0, 1–4, 5–9, 10–19, and 20+), with grey indicating missing data. Most regions have low to moderate numbers of studies, with higher concentrations in parts of England, particularly the South East, while Scotland, Wales, and Northern Ireland show fewer studies overall.


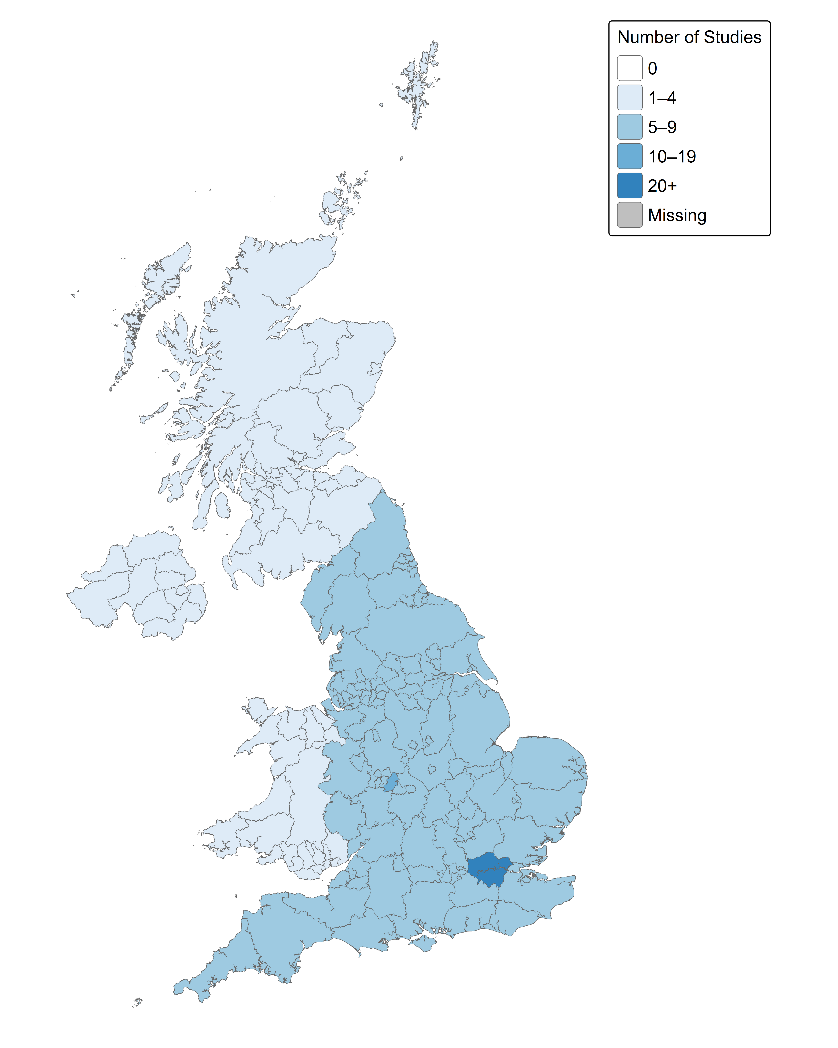


**Figure S2** Heat map summarising prevalence of infection/test positivity for IGRA test yield, HIV, HBV and HCV infection (%, (x/n)) and active TB (prevalence per 100,000, (x/n)), stratified by sex/gender and age. Multiple columns for an infection represent findings from independent studies. Further details of included studies (1-6) are shown in Supplementary Material Tables S5-10, which also includes prevalence and test yield estimates for other risk factors.

Colour scheme represents the categorisation described in the Methods section. HIV: red = extremely high prevalence (>0.5%), orange = high (0.2–0.5%), green = low (<0.2%) (7, 8). HBV: red = high prevalence (≥5%), orange = intermediate (2-4.9%), green = low (<2%) (9). HCV: red = high prevalence (>0.36%), orange = intermediate (>0.18-0.36%), green = low (≤0.18%). IGRA: red = high yield (>15%), orange = intermediate (>7.5-15%), green = low (≤7.5%). Active TB: red = extremely high prevalence (≥150 per 100,000 population), orange = high (40-149 per 100,000), green = low (<40 per 100,000) (10). Multiple estimates within one cell have not been pooled because of their heterogeneity. However, simple pooling of estimates was conducted to determine colour categories for these cells.

**Figure S2 alt text** Graphical representation of prevalence of infection and interferon gamma release assay (IGRA) test yield estimates, shown by sex/gender and age of migrants tested.

**Figure S2**

**Figure S3** Scatterplot illustrating the association between study sample size and number of risk factors for infection reported by included studies. Menezes et al (11), Aldridge et al (3) and Zenner et al (12) are shown as a single point (sample size 2,006,671) with total risk factors reported across all three papers, as they report from the same dataset. Severi et al (13) reported active TB prevalence stratified by four risk factors, but the outcome reported was prevalence of active TB cases subsequently reported to the Enhanced TB Surveillance System rather than the number of migrants identified as suspected of having active TB at the time of screening.

**Figure S3 alt text** Scatterplot showing the relationship between study sample size (x-axis) and the number of infection risk factors reported (y-axis) across included studies. Each point represents a study. More than half the scatter points lie on the x-axis and there is no obvious relationship between study sample size and number of risk factors reported for the remaining points.

**Figure S4** Relationship between numbers of migrants in UK by country of birth for most frequent non-EU countries, and TB incidence and HIV, HBV, HCV prevalence in country of birth. Underlined text on the right hand side shows the number of country-specific prevalence estimates included in our risk factor analysis (Figure 4, main manuscript), reported for migrants from each country, for each infection. * One HBV estimate reported from mainland China and one reported from Hong Kong. TB incidence estimates from World Health Organization (WHO) TB profiles (14); HIV prevalence among 15-49-year-olds estimates from the WHO Global Health Observatory (15) and UNAIDS country summaries (16); HBV surface antigen (HBsAg) prevalence estimates from the WHO Global Health Observatory (15); HCV estimates from The Coalition for Global Hepatitis Elimination Data and Profiles (17). Overseas-born population in the UK by country of birth 2020-2021 from the UK Office for National Statistics (18).

**Figure S4 alt text** Horizontal bar and scatter plot showing the relationship between the size of overseas-born populations in the UK by country of birth (bars) and infection burden in countries of birth (points). Grey bars represent overseas-born population size, while symbols indicate TB incidence and HIV, HBV, and HCV prevalence. Countries are listed on the y-axis, with higher migrant populations generally corresponding to countries with varying levels of TB and blood-borne virus prevalence. Underlined annotations indicate the number of country-specific prevalence estimates included in the risk factor analysis for each infection.

2 HBV, 2 HCV

2 HBV*

1 active TB, 2 HBV, 2 HCV

1 active TB, 3 HBV, 2 HCV

1 active TB

1 active TB

1 active TB, 1 HIV, 2 HBV, 2 HCV

1 latent TB, 1 HIV, 2 HBV, 2 HCV

**Figure S5** Scatter plot of infection prevalence by country of origin of UK migrants against infection prevalence in country of origin, for HIV, HBV and HCV. Error bars represent 95% confidence interval estimates for migrant prevalence for countries included in the risk analysis, shown in Figure 4, calculated using the Wilson score method. Infection prevalence in country of origin is from UNAIDS for HIV (% of population aged 15-49, summarised by The World Bank (19)) and the Coalition for Global Hepatitis Elimination website (HBV, defined as prevalence of chronic HBV (HBsAg+) and HCV, defined as prevalence of chronic HCV (RNA+/cAg) (20)). (Estimate of 40.8% prevalence of chronic HCV (RNA+/cAg) for India has not been used – suspected typographical error.) The dashed diagonal line indicates equality between origin and migrant prevalence.

**Figure S5 alt text** Scatter plot comparing infection prevalence in migrants to the UK (y-axis) with infection prevalence in their countries of origin (x-axis), both shown as percentages. Each point represents a country–infection combination and is coloured by infection type. Vertical error bars indicate confidence intervals around migrant prevalence estimates.


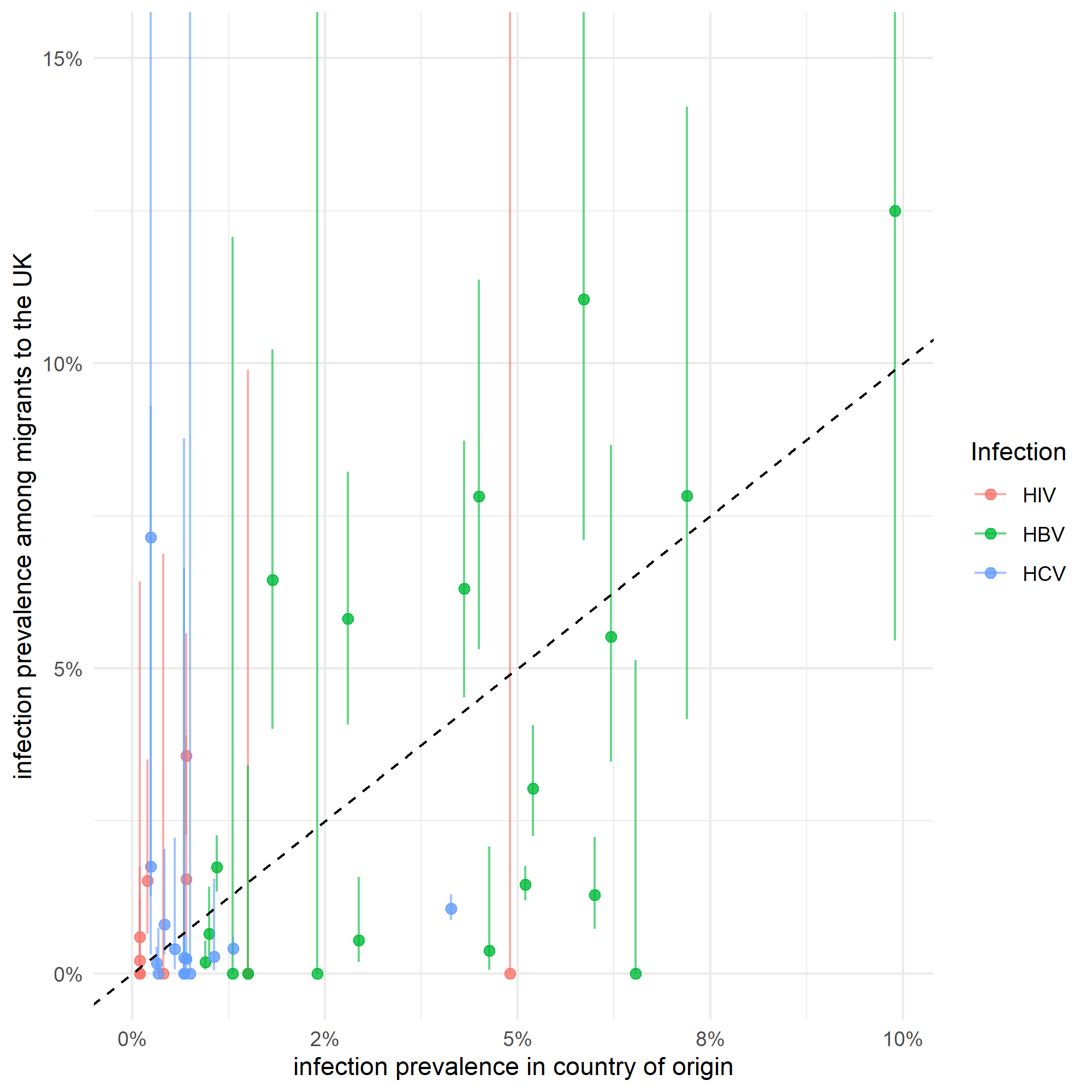


**Table S1** Summary of included studies with test yields for IGRA and prevalence of active TB, HIV, HBV and HCV infections.

| **Author, year** | **Study location** | **Years of data collection** | **Study design and population** | **Age*** | **% female*** | **Countries/ regions of birth/origin** | **Prevalence of infection (%, x/n)** | | | | |  | |
| --- | --- | --- | --- | --- | --- | --- | --- | --- | --- | --- | --- | --- | --- |
|  |  |  |  |  |  |  | **IGRA test yield/ LTBI** (%, x/n)** | **Active TB (per 100,000, x/n)** | **HIV**  **(%, x/n)** | **HBV*** (%, x/n)** | **HCV†**  **(%, x/n)** | |  |
| Harling 2007 (21) | England (Dover) | 2002 | Cross-sectional descriptive study of asylum seekers managed through the Dover Induction Centres. | Mean 24.6y, SD 9.7y | 20% | 16% Europe, 11% Africa, 61% E Mediterranean, 1% SE Asia, 1% W Pacific‡ | –††† | 70, 3/4275  (symptoms and/or Heaf reaction plus CXR) | – | – | – | |  |
| Burns 2009 (22) | London | 2006-2007 | Cross-sectional study of new attendances at GUM clinics who were migrants from eight central and eastern European countries. | Med 27y, IQR 24-31y (men); med 25y, IQR 22-28y (women) | 77% | 45% Poland, 18% Lithuania, 11% Slovakia, 11% Czechia, 7% Hungary, 6% Latvia, 2% Estonia, 1% Slovenia | – | – | 0.18%, 3/1701 | – | – | |  |
| Uddin 2010 (5) | London, Walsall, Sandwell, Bradford | NS | Community-based testing project for migrants at 52 sites in five regions in England. Migrants recruited through local religious leaders and community representatives attending community centres for screening. | NS | NS | 27% India, 17% Bangladesh,  56% Pakistan | – | – | – | 1.28%, 56/4381 | 1.67%, 73/4381 | |  |
| Platt 2011 (23) | London | 2008-2009 | Cross-sectional survey of female sex workers, reporting results for migrant participants only. | Med 25y, IQR 22-29y | 100% | E Europe and the Former Soviet Union | – | – | 1.23%, 2/163 | – | – | |  |
| Pareek 2011 (2) | London, Leeds, Blackburn | 2008-2010 | Multicentre study of foreign-born new UK entrants (arrival 15y) referred for TB screening through port-of-entry screening systems, health protection units, or after registration with primary-care services. | 3% ≤16y,  48% 16-25y, 49% 26-35y | 51% | Most common regions: Indian subcontinent, Sub Saharan Africa. Most common countries: 32% Pakistan, 26% India | 19.97%, 245/1227 | – | – | – | – | |  |
| Pareek 2013 (24) | London | 2008-2010 | Observational study: prospective assessment of screening recent migrants (<5y) from high TB incidence countries, comparing TB diagnostic tests, undertaken in GP clinics. | 37.7% 16-25y, 36.4% 26-35y, 15.2% 36-45y, 10.8% >45y | 65% | 7% Europe/N America, 6% S America, 8% Middle East, 2% Other Africa, 42% Other Asia, 21% Indian subcontinent, 13% SSA | 16.59%, 38/229 | – | – | – | – | |  |
| Vedio 2013 (25) | Sheffield | 2009-2012 | Cross-sectional study screening Chinese residents through testing events advertised and implemented in a community setting. | Med 47y, IQR 33-55y, range 15-86y | 55% | China | – | – | – | 8.93%, 20/224 | 0.00%, 0/224 | |  |
| Mcpherson 2013 (26) | NE England | NS | Cross-sectional study inviting members of British–Chinese and British–South Asian communities to attend HBV education and screening in community settings. | NS | NS | 54% British–Chinese, 46% British–South Asian | – | – | – | 5.82%, 57/980 | – | |  |
| O'Leary 2013 (27) | Glasgow | 1993-2010 | Cross-sectional, community-based survey of migrants recruited from six mosques and four community centres. | NS | NS | 79% Pakistan, 12% India, 9% Other | – | – | – | – | 1.63%, 21/1288 | |  |
| Balogun 2013 (28) | England (Bury & Rochdale (Greater  Manchester), Birmingham (West Midlands), Camden  & Islington (north London) and Enfield (north London)) | 2001-2005 | Cross-sectional study recruiting non-UK-born children through primary schools. | 7-11y | NS | Only ethnicity described: 18% Black African, 18% White, 12% Pakistani, 10% Black Caribbean, 8% Bangladeshi, 5% Indian, 28% Other | – | – | – | 0.14%, 1/729 | – | |  |
| Sidebottom 2014 (abstract) (29) | Huddersfield | 2011-2012 | Cross-sectional pilot study evaluating HIV testing at induction centres for asylum seekers. | NS | NS | Countries with >1% HIV prevalence | – | – | 7.5%, 14/187 | – | – | |  |
| O'Shea 2014 (30) | England | 2012 | Cohort study comparing IGRA and TST tests for diagnosing LTBI in Nepalese (Ghurka) recruits to the British Army recently arrived in the UK. Results are IGRA test positivity combining tests performed on days 0 and 7 of testing. | 18-21y | 0% | Nepal | 17.47%, 29/166 | – | – | – | – | |  |
| Mc Grath-Lone 2014a (31) | England (primarily London) | 2011 | Cross-sectional study: analysis of EHRs for migrant female sex worker GUM clinic attendees across England. | Med 28y  4.1% ≤19y, 26.5%  20-24y, 25.9% 25-29y, 19.8% 30-34y, 19.0% 35-44y, 4.6% 45+y | 100% | 47% E Europe, 26% S America,  16% Asia,  5% Europe (exc UK and E Europe), 4%  Africa, 1%  N America, 1% Australia | – | – | 0.26%, 4/1511 | – | – | |  |
| Mc Grath-Lone 2014b (32) | England | 2011 | Cross-sectional study: analysis of EHRs for migrant male sex worker GUM clinic attendees across England. | NS | 0% | 39% S America (97% of these from Brazil), 25% Europe, 12% E Europe | – | – | 6.92%, 9/130 | – | – | |  |
| Hargreaves 2014 (33) | London | 2013 | Cross-sectional study of new migrants attending a New Patient Health Check in 2 GPs attached to A&E departments. | 33y, range 18–72y | 53% | 63% Asia, 17% Africa, 9% Latin America, 11% E Europe | 18.18%, 6/33 | – | 0.00%, 0/36 | 0.00%, 0/36 | 0.00%, 0/36 | |  |
| Cochrane 2015 (34) | Bristol | 2006-2014 | Retrospective HER data linkage review of antenatal HBV testing data for women born in high/ intermediate (>2%) HBV prevalence countries. | NS | 100% | HBV prevalence >2%. Africa, Asia and Pacific, Latin America and Caribbean, E and S Europe | – | – | – | 1.73%, 101/5840 | – | |  |
| Evlampidou 2016 (35) | Bristol | 2006-2013 | Cross-sectional database linkage study of migrants tested for HBV, registered with a GP and born in a high HBV prevalence country (≥2%). | 1% <18y, 6% 18-24y, 41% 25-34y, 37% 35-44y, 9% 45-54y, 33% 55-64y, 2% >65y | 75% | HBV prevalence ≥2%.  25% E Africa, 1% Middle Africa, 2% N Africa, 3% Southern Africa, 6% W Africa, 4% E Asia, 20% S Asia, 5% SE Asia, 4% W Asia, 5% Caribbean, 2% S America, 16% E Europe, 7% S Europe | – | – | – | 4.75%, 457/9627 | – | |  |
| Aldridge 2016 (3)†† | England, Wales, N Ireland | 2005-2013 | Population-based cross-sectional pilot study of a pre-entry TB screening programme of applicants for long-term UK visas in 15 countries with high TB incidence. Screened at health centres in country of origin. | 4% 11-18, 94% 16-44y, 2% 45-64, 1% ≥65y | 35% | High TB incidence countries (>40/100,000): Bangladesh, Burkina Faso, Cambodia,  Côte d’Ivoire, Eritrea, Ghana, Kenya, Laos, Niger,  Pakistan, Somalia, Sudan, Tanzania, Thailand, Togo | – | 92, 438/476,455 (bacteriologica-lly confirmed) ¶¶ | – | – | – | |  |
| Severi 2016 (13) | London airports | 2009-2010 | Cross-sectional study reporting results of the airport screening programme (new UK entrants). | Med 25y, IQR 22-29y  43.6% 16-24y, 34.4% 25-29y, 20.7% 30-54y,  1.3% 55+y | 43% | NS | – | 339, 678/200,199  (medical assessment, may include CXR) | – | – | – | |  |
| Usdin 2017 (36) | Birmingham | 2014 | Pilot study of effectiveness of LTBI screening for new migrants in a community college delivered on campus. | 28% 15-20y, 15% 21-25y, 28% 16-30y, 30% 31-35y | 54% | High TB incidence countries | 16.14%, 71/440 | 455, 2/440  (symptoms) | – | – | – | |  |
| Crawshaw 2018 (37) | UK | 2013-2017 | Cross-sectional prevalence study among refugees. Data collection from the International Organisation for Migration clinics enrolled in the UK pre-entry migration health assessments. | 15-49 | 49% | 91% E Mediterranean, 9% African. | – | 92, 9/9759  (symptoms, CXR, culture, smear) | 0.38%, 31/8056 | 2.04%, 188/9228 | 0.41%, 38/9223 | |  |
| Abubakar 2018 (38) | London, Birmingham, Leicester | 2010-2015 | Baseline data from prospective cohort (comparison of TB diagnostic tests). New UK entrants (<5y). | Med 33y, IQR 26-51y | 50% | High TB incidence countries§ | 22.09%, 940/4256^#^ | – | – | – | – | |  |
| Flanagan 2019 (4) | Bradford, Yorkshire, London | 2013-2017 | Multicentre, cluster-randomised controlled trial in GPs in areas with high migrant density, trialling interventions to improve rates of screening for viral hepatitis. | 2% 18-19y, 19% 20-29y, 26% 30-39y, 23% 40-49y, 15% 50-59y, 10% 60-69y, 7% ≥70y | 57% | “High HBV/HCV risk countries”.  Ethnicity: 6%  Black, 8% Bangladeshi, 10% Indian, 57% Pakistani, 3% Other Asian, 3%  E European, 12% Other | – | – | – | 1.06%, 127/11929 | 0.30%, 36/11929 | |  |
| Berrocal-almanza 2019 (39) | England | NS | Restrospective, population-based cohort study. New entrants to the UK who were negative for active TB and were eligible for LTBI screening. | NS | NS | NS | 17.18%, 421/2451 | – | – | – | – | |  |
| Hargreaves 2020 (40) | UK | NS | Feasibility study of screening recent migrants (<10y since UK entry) in A&E. | Mean 35y, SD 12y, range 18–73y | 48% | 29% E Europe/former S Union, 4%  Central/Southern Europe, 20%  Middle East, 25% Africa, 10% S America, 12% Asia | 14.58%, 14/96 | – | 1.04%, 1/96 | 2.08%, 2/96 | – | |  |
| Kelly 2020 (41) | SE England | 2018 | Prospective descriptive feasibility study assessing the effectiveness of an interventional film in promoting testing for HCV for South Asian first-generation migrants. | 4% 18-24y, 11% 25-40y, 9% 41-50y, 12% 51-60y, 64% ≥61y | 50% | 47% India, 39% Pakistan, 11% Bangladesh, 3%  Other South Asian¶ | – | – | – | 0.95%, 2/211 | 0.47%, 1/211 | |  |
| Menezes 2022 (11) | UK | 2005-2018 | Cross-sectional. Comparison of four national migrant TB screening programmes (presenting results for UK only – UK new entrants, from high TB incidence countries: >40/100,000). | “Mostly young adults” | 55% | Most common countries: 18% Pakistan, 16% Philippines, 15% Thailand | – | 69, 1383/2,006,671  (symptoms, CXR) | – | – | – | |  |
| Pinto 2022 (abstract) (42) | London | 2019-2021 | Retrospective analysis of GHRs for unaccompanied asylum seeking children attending "one-stop shop" hospital-based screening programme. | Med 17y, IQR 16-17y | 11% | 3 most frequent countries: 26% Sudan, 23% Eritrea, 17% Afghanistan | 21.01%, 25/119‡‡ | 1681, 2/119 (diagnostic method NS) | 1.68%, 2/119 | 4.20%, 5/119 | 0.00%, 0/117 | |  |
| Berrocal-almanza 2022 (43) | High-TB burden regions of England | 2011-2018 | Retrospective, population-based cross-sectional study assessing the effectiveness of the national primary care-based migrant LTBI testing programme. Testing criteria: migrants 16-35y from high TB-incidence countries (≥150 cases/ 100,000/y or any country in SSA), entered England <5y, registering with primary care | 16-35y  44% 16-25y, 55% 26-35y | 45% | 77% S Asia, 19% Africa, 3% E & SE Asia,  1% The Americas and Europe | 18.18%, 6640/36,532 | – | – | – | – | |  |
| Zenner 2023 (12)†† | UK | 2005-2018 | Cross-sectional. Comparison of four national migrant TB screening programmes (presenting results for UK only – UK new entrants from high TB incidence countries: >40/100,000). | NS | NS | NS | – | 69, 1383/2,006,671  (symptoms, CXR) | – | – | – | |  |
| Eisen 2023 (44) | London | 2016-2022 | Retrospective study of unaccompanied asylum-seeking children attending for testing in 3 clinics in London | Med 16y, range 11-18y. 84% 16-18y, 3% <14y | 11% | 20% Eritrea, 18% Afghanistan, 15% Sudan, 9%  Iran, 9% Vietnam, 7% Albania, 6% Ethiopia, 4% Iraq, 2% Syria, 8% Other | 17.94%, 193/1076 | – | 0.19%, 2/1071 | 3.94%, 42/1067 | 0.09%, 1/1069 | |  |
| Kelly 2023 (45) | Hampshire, Surrey, Berkshire | NS | Cross-sectional results from a mixed methods prospective community-based cohort study exploring awareness and prevalence of chronic viral hepatitis in a UK-based Nepali population. | Median 66y, IQR 60-70y, range 19-86y | 48% or 52%§§§ | Vast majority from Nepal | – | – | – | 0.30%, 3/994 | 0.00%, 0/994 | |  |
| Amrose 2023 (abstract) (46) | London | 2020-2022 | Cross-sectional retrospective review of EHRs of asylum seekers attending sexual health services or undergoing routine BBV screening in A&E. | NS | NS | NS | – | – | 6.48%, 7/108 | – | – | |  |
| Barry 2023 (1) | Wales | 2022 | Cross-sectional. Routine data from national screening programme for Ukrainian refugees. | Med 30y, IQR 14-41y. 33% <18y | 66% | Ukraine | 6.86%, 111/1617^##^ | 62, 1/1617^##^  (diagnostic algorithm using available data on symptoms, CXR, IGRA test, mycobacterial culture) | – | – | – | |  |
| Martyn 2024 (47) | London | 2020-2022 | Cross-sectional study; evaluation of an outreach, point-of-care screening programme for asylum seekers recruited from initial accommodation centres (IACs). | Adults | NS | NS | – | – | – | 3.46%, 28/809 | – | |  |
| Baggaley (6) | Leicester | 2016-2019 | Retrospective observational study. Analysis of laboratory and infectious disease clinic records linked to a migrant status indicator. | 16–65y  32% 16–25y, 41% 26–35y,  15% 36–45y,  8% 46–55y,  4% 56–ss65y  0.4% 66+y | 53% | NS | 19.38%, 496/2560‡‡‡ | 664 (17/2560) ‡‡‡ | 0.48%, 17/3545 | 3.34%, 117/3502 | 0.18%, 6/3402 | |  |

A&E = Accident & Emergency (hospital Emergency Room); BBV – blood-borne virus; CXR = chest X-ray; E = east; EHRs = electronic health records; exc = excluding; GP = general practices; GUM = Genitourinary Medicine; IAC = temporary accommodation centre for people seeking asylum; IQR = interquartile range; LTBI = latent tuberculosis infection; med = median; NE = northeast; NS = not stated; S = south; SD = standard deviation; SE = southeast; SSA = Sub Saharan Africa; TB = tuberculosis; y = years; – = not measured.

* Percentages calculated exclude study participants with missing data.

** The IGRA test measures likelihood of past exposure to TB and suggests latent TB infection (LTBI). The majority of recent studies use the IGRA test to estimate LTBI prevalence but other tests e.g., TST may still be used. Unless otherwise stated, all included studies used IGRA.

*** Hepatitis B surface antigen (HBsAg) test indicating current HBV infection.

† Test for active HCV infection (RNA test). Studies reporting HCV antibody test yield (indicating a history of HCV exposure rather than active infection) are excluded (results shown in Tables S1 and S2, Supplementary Material).

‡ Data from the whole year of follow-up, while prevalence data are from the first 6 months of follow-up only.

§ Inclusion criteria included being new entrants to the UK, but 129 (2.7%) of the study population were reported as UK-born.

¶ Of the 184 migrants tested who watched an intervention film; no data on country of birth/origin was recorded for the remaining 45 (20%) who were recruited for testing by word of mouth.

# Number of participants and those testing positive at baseline was not stated for all three diagnostic tests combined. Therefore data presented are for the QFT-GIT IGRA (QuantiFeron®-TB Gold In-Tube) test, which tested the most new-UK entrants at baseline.

†† Not included in the forest plot analysis because overall prevalence estimates are reported by Menezes et al (11); however Aldridge et al 2016 (3) and Zenner et al 2023 (12) both provide additional prevalence data stratified by risk factors.

‡‡ Type of test for latent tuberculosis infection not stated.

§§ Despite similarities of study location and data collection years, the corresponding author states that Pareek et al 2013 (24) and Pareek et al 2011 (2) report independent data.

¶¶ Crude prevalence of clinically diagnosed cases, excluding laboratory confirmed cases, was 3 (2–4) per 100,000. Number of active TB cases derived using the prevalence estimate and sample size.

## Denominator amended from 1955 (as stated in Barry et al 2023 (1)) to 1617 (Gareth Davies, personal communication), which excludes <11 year olds from the analysis as only high risk children (symptomatic or close contacts of active TB cases) were tested, in accordance with TB screening guidelines.

††† Harling et al 2007 (21) additionally reported that 93 of 4275 subjects screened positive for TB based on a positive Heaf reaction and/or symptoms, but this information has been excluded because for half of the study period, Heaf tests were only administered to participants without a BCG scar.

‡‡‡ Of the 496 testing IGRA positive, 437 (88%) attended clinic, of whom 17 (4%) were diagnosed with active TB infection; 14 (3%) were diagnosed with previously-treated active TB infection; 403 (92%) were diagnosed with latent TB infection; and three (0.7%) were lost to follow-up. To be consistent with the reported outcomes from other studies, which report test yield at the time of screening, we report IGRA positivity without accounting for subsequent active TB diagnoses.

§§§ 525 male and 480 female stated in abstract but 525 females stated in the Results section.

**Table S2:** Summary of excluded studies reporting history of HCV infection (HCV antibody test).

| **Author, year** | **Study location** | **Years of data collection** | **Study design and population** | **Age*** | **% female*** | **Countries/ regions of birth/origin** | **Prevalence of history of exposure to HCV (%, x/n)** |
| --- | --- | --- | --- | --- | --- | --- | --- |
| Cortina-Borja 2016 (48) | England | 2012 | Cross-sectional study. Analysis of neonatal dried blood spot samples to neonates born to non-UK-born pregnant mothers. | 2% <21y, 18% 21-25y, 35% 26-30y, 26% 31-35, 19% >35y | 100% | 22% Africa, 31% Europe (exc UK), 6% Americas, 41% Asia-Pacific regions | 0.16%, 23/14,346 |
| Hargreaves 2020 (40) | UK | NS | Feasibility study of screening recent migrants (<10y since UK entry) in A&E. | Mean 35y, SD 12y, range 18–73y | 48% | 29% E Europe/former S Union, 4%  Central/Southern Europe, 20% Middle East, 25% Africa, 10% S America, 12% Asia | 1.04%, 1/96 |

* Percentages calculated exclude study participants with missing data.

**Table S3:** Summary of quality assessment of included studies using the Joanna Briggs Quality Assessment Tool (49). See footnotes for complete wording for each criterion.

|  | Sample frame | Study participants | Sample size | Subjects & setting | Data analysis | Methods validity | Condition measurement | Statistical analysis | Response rate | Overall appraisal* |
| --- | --- | --- | --- | --- | --- | --- | --- | --- | --- | --- |
| Harling 2007 (21) | Yes | Yes | Yes | Yes | Yes | No | Yes | Yes | Yes | Low |
| Burns 2009 (22) | Yes | Yes | Yes | Yes | Yes | Yes | Yes | Yes | Yes | High |
| Uddin 2010 (5) |  | Yes | Yes | Yes | Yes | Yes | Yes | Yes | Yes | High |
| Platt 2011 (23) | Yes | Yes | Yes | Yes | Yes | Yes | Yes | Yes | Yes | High |
| Pareek 2011 (2) | Yes | Yes | Yes | Yes | Yes | Yes | Yes | Yes | Yes | High |
| Pareek 2013 (24) | Yes | Yes | Yes | Yes | Yes | Yes | Yes | Yes | Yes | High |
| Vedio 2013 (25) | Yes | Yes | Yes | Yes | Yes | Yes | Yes | Yes | Yes | High |
| Mcpherson 2013 (26) | Yes | Yes | Yes | Yes | Yes | Yes | Yes | Yes | Yes | High |
| O'Leary 2013 (27) | Yes | Yes | Yes | Yes | Yes | Yes | Yes | Yes | Yes | High |
| Balogun 2013 (28) | Yes | Yes | Yes | Yes | Yes | Yes | Yes | Yes | Yes | High |
| Sidebottom 2014 (abstract) (29) | Yes | Yes | Yes | Yes | Yes | Yes | Yes | Yes | Yes | Low |
| O'Shea 2014 (30) | Yes | Yes | Yes | Yes | Yes | Yes | Yes | Yes | Yes | High |
| Mc Grath-Lone 2014a (31) | Yes | Yes | Yes | Yes | Yes | Yes | Yes | Yes | Yes | High |
| Mc Grath-Lone 2014b (32) | Yes | Yes | Yes | No | Yes | Yes | Yes | Yes | Yes | Moderate |
| Hargreaves 2014 (33) | Yes | Yes | No | Yes | Yes | Yes | Yes | Yes | No | Moderate |
| Cochrane 2015 (34) | Yes | Yes | Yes | Yes | Yes | Yes | Yes | Yes | Yes | High |
| Evlampidou 2016 (35) | Yes | Yes | Yes | Yes | Yes | Yes | Yes | Unclear | Yes | Moderate |
| Aldridge 2016 (3) | Yes | Yes | Yes | Yes | Yes | Yes | Yes | Yes | Yes | High |
| Severi 2016 (13) | Yes | Yes | Yes | Yes | Yes | Unclear | Unclear | Yes | Yes | Moderate |
| Usdin 2017 (36) | Yes | Yes | Yes | Yes | Yes | Yes | Yes | Yes | Yes | High |
| Crawshaw 2018 (37) | Yes | Yes | Yes | Yes | Yes | Yes | Yes | Yes | Yes | High |
| Abubakar 2018 (38) | Yes | Yes | Yes | No | Yes | Yes | Yes | Yes | Yes | High |
| Flanagan 2019 (4) | Yes | Yes | Yes | Yes | Yes | Yes | Yes | Yes | Yes | High |
| Berrocal-almanza 2019 (39) | Yes | Yes | Yes | No | Yes | Yes | Yes | Yes | Yes | High |
| Hargreaves 2020 (40) | No | Yes | No | Yes | No | Yes | Yes | Yes | No | Moderate |
| Kelly 2020 (41) | Yes | Yes | Yes | Yes | Yes | Yes | Yes | Yes | Yes | High |
| Menezes 2022 (11) | Yes | Yes | Yes | No | Yes | Yes | Yes | Yes | Yes | High |
| Pinto 2022 (abstract) (42) | Yes | Yes | Yes | No | Yes | Unclear | Unclear | Unclear | Yes | Low |
| Berrocal-almanza 2022 (43) | Yes | Yes | Yes | Yes | Yes | Yes | Yes | Yes | Yes | High |
| Zenner 2023 (12) | Yes | Yes | Yes | Yes | Yes | Yes | Yes | Yes | Yes | High |
| Eisen 2023 (44) | Yes | Yes | Yes | Yes | Yes | Yes | Yes | Yes | Yes | High |
| Kelly 2023 (45) | Yes | Yes | Yes | Unclear | Yes | Yes | Yes | Yes | Yes | Moderate |
| Amrose 2023 (abstract) (46) | No | Yes | No | No | Yes | Yes | Yes | Yes | Yes | Low |
| Barry 2023 (1) | Yes | Yes | Yes | Yes | Yes | Yes | Yes | No | Yes | High |
| Martyn 2024 (47) | Yes | Yes | Yes | No | Yes | Yes | Yes | Yes | Yes | Moderate |
| Baggaley et al 2025 (6) | Yes | Yes | Yes | Yes | Yes | Yes | Yes | Yes | Yes | High |

Q1: Was the sample frame appropriate to address the target population? Q2: Were study participants sampled in an appropriate way? Q3: Was the sample size adequate? Q4: Were the study subjects and the setting described in detail? Q5: Was the data analysis conducted with sufficient coverage of the identified sample? Q6: Were valid methods used for the identification of the condition? Q7: Was the condition measured in a standard, reliable way for all participants? Q8: Was there appropriate statistical analysis? Q9: Was the response rate adequate, and if not, was the low response rate managed appropriately?

Given the inevitable limited detail available in conference abstracts, the overall appraisal score for these studies has been assigned to be low. Zenner et al 2023 (12) and Aldridge et al 2016 (3) were not included in the primary analysis because they report the same data as Menezes et al 2022 (11), but are included in the quality assessment because they are included in the risk factors analysis.

* High, moderate or low.

**Table S4:** Coinfection prevalence reported by studies reporting prevalence estimates for multiple infections*.

| **Study** | **Tests used** | **Prevalence of coinfection, % (95%CI), x/n** |
| --- | --- | --- |
| Uddin 2010 (5) | HBV, HCV | **0.09%** (0.04-0.23%), 4/4381 **HBV-HCV*** |
| Hargreaves 2014 (33) | IGRA, HIV, HBV, HCV | **0.00%** (0.00-9.64%), 0/36 **IGRA-HIV-HBV-HCV**** |
| Crawshaw 2018 (37) | Active TB, HIV, HBV, HCV | Assumed 0.00% coinfections involving TB and HCV.  **0.02%** (0.01-0.09%), 2/8056 **HIV-HBV***** |
| Flanagan 2019 (4) | HBV, HCV | **0.01%** (0.00-0.05%), 1/11,929 **HBV-HCV** |
| Hargreaves 2020 (40) | IGRA, HIV, HBV | **1.04%** (0.18-5.67%), 1/96 **IGRA-HBV**†  **0.00%** (0.00-3.85%), 0/96 **IGRA-HIV**, **HIV-HBV** |
| Amrose 2023 (abstract) (46) | HIV, HBV | **1.85%** (0.51-6.50%), 2/108 **HIV-HBV** |
| Kelly 2023 (45) | HBV, HCV | **0.00%** (0.00-0.385%), 0/994 **HBV-HCV** |
| Baggaley 2025 (6) | IGRA, HIV, HBV, HCV | **0.06%** (0.02-0.21%), 2/3475 **HIV-HBV**  **0.14%** (0.05-0.41%), 3/2128 **IGRA-HIV**  **0.80%** (0.50-1.30%), 17/2124 **IGRA-HBV**  No coinfections with HCV; no multiple infections with >2 infections |

Eisen 2023 (44) reported that, “116 (11%) had multiple infections” but this was excluded because this included infections other than TB, HIV, HBV and HCV e.g., schistosomiasis.

* Coinfection prevalence stratified by country: Bangladesh 0.00% (95%CI 0.00-0.53%), 0/726; India 0.00% (95%CI 0.00-0.32%), 0/1197; Pakistan 0.16% (95%CI 0.06-0.42%), 4/2458.

** 0.00% coinfection prevalence for all infection combinations because prevalence of HIV, HBV and HCV in the study sample was 0.00%

*** Crawshaw et al 2018 (37) reported that two participants with HIV-hepatitis B coinfections were identified. We therefore assumed no coinfections involving TB and HCV. 8056 study participants were tested for HIV and 9228 for HBV; we estimate coinfection prevalence assuming 8056 participants were tested for both infections, while acknowledging that this is likely to be an overestimate, and therefore our derived estimate of HIV/HBV coinfection prevalence is likely to be a slight overestimate. No participant had >2 concurrent infections.

† Hargreaves 2020 (40) reported that one patient who screened IGRA test positive also tested positive for HBV. We therefore assumed no coinfections involving HIV.

**Table S5:** Prevalence of IGRA positivity stratified by risk factors, reported by included studies. Studies are arranged with equivalent/similar reported risk factors across the same row, to facilitate comparison (where studies stratify by similar but not identical risk factors, the precise risk factor used for each study is shown in brackets under “Study”).

| **Risk factor** | **Categories** | **%** | **x/n** | **Categories** | **%** | **x/n** | **Categories** | **%** | **x/n** | **Categories** | **%** | **x/n** |
| --- | --- | --- | --- | --- | --- | --- | --- | --- | --- | --- | --- | --- |
|  | **Pareek 2013 (24)*** |  |  | **Pareek 2011 (2)** |  |  | **Baggaley 2025 (6)** | | | **Barry 2023 (1)**** | |  |
| Sex/gender*** | Women | 15.65% | 23/147 | Female | 17.33% | 109/629 | Female | 15.25% | 227/1489 | Female | 5.61% | 65/1159 |
|  | Men | 18.29% | 15/82 | Male | 22.67% | 136/600 | Male | 25.12% | 269/1071 | Male | 8.48% | 47/554 |
| Age (years) | 16-25 | 13.95% | 12/86 | <16 | 19.44% | 7/36 | 16-25 | 15.18% | 172/1133 | 11-17† | 1.68% | 5/298 |
|  | 26-35 | 11.90% | 10/84 | 16-25 | 14.60% | 86/589 | 26-35 | 22.53% | 319/1416 | 18-29 | 2.90% | 9/310 |
|  | 36-45 | 23.53% | 8/34 | 26-35 | 25.17% | 152/604 | 36-45 | 40.00% | 2/5 | 30-39 | 7.92% | 35/442 |
|  | >45 | 32.00% | 8/25 |  |  |  | 46-55 | 66.67% | 2/3 | 40-49 | 9.94% | 33/332 |
|  |  |  |  |  |  |  | 56-65 | 50.00% | 1/2 | 50-64 | 12.96% | 21/162 |
|  |  |  |  |  |  |  | ≥66 | 0.00% | 0/1 | ≥65 | 10.96% | 8/73 |
|  | **Pareek 2013 (24)*** |  |  | **Pareek 2011 (2)** |  |  | **Baggaley 2025 (6)** | | | **Eisen 2023 (44)** |  |  |
| World region of | Europe, Americas | 0.00% | 0/30 | Europe, Americas | 4.00% | 2/50 |  |  |  | Afghanistan | 15.03% | 29/193 |
| origin/country of | Middle East, N Africa | 8.33% | 2/24 | Middle East, N Africa | 3.85% | 1/26 |  |  |  | Albania | 2.78% | 2/72 |
| Birth | Other Asia | 20.83% | 20/96 | Other Asia | 17.90% | 29/162 |  |  |  | Eritrea | 28.64% | 63/220 |
|  | Indian subcontinent | 14.29% | 7/49 | Indian subcontinent | 19.46% | 144/740 |  |  |  | Ethiopia | 45.76% | 27/59 |
|  | Sub Saharan Africa | 30.00% | 9/30 | Sub Saharan Africa | 27.49% | 69/251 |  |  |  | Iran | 1.10% | 1/91 |
|  |  |  |  |  |  |  |  |  |  | Iraq | 2.70% | 1/37 |
|  |  |  |  |  |  |  |  |  |  | Sudan | 27.33% | 44/161 |
|  |  |  |  |  |  |  |  |  |  | Syria | 0.00% | 0/25 |
|  |  |  |  |  |  |  |  |  |  | Vietnam | 5.38% | 5/93 |
| TB incidence in country of origin (per 100,000 per annum) | ≤65 | 5.13% | 2/39 | 0-50 | 3.13% | 1/32 |  |  |  |  |  |  |
|  | 66-170 | 11.65% | 12/103 | 51-150 | 12.67% | 19/150 |  |  |  |  |  |  |
|  | 171-300 | 29.73% | 22/74 | 151-250 | 19.64% | 164/835 |  |  |  |  |  |  |
|  | >300 | 15.38% | 2/13 | 251-350 | 29.50% | 41/139 |  |  |  |  |  |  |
|  |  |  |  | >350 | 27.40% | 20/73 |  |  |  |  |  |  |
| Ethnicity |  |  |  |  |  |  | Indian | 22.24% | 266/1196 |  |  |  |
|  |  |  |  |  |  |  | Pakistani | 19.48% | 15/77 |  |  |  |
|  |  |  |  |  |  |  | Bangladeshi | 14.29% | 9/63 |  |  |  |
|  |  |  |  |  |  |  | Other Asian | 12.01% | 46/383 |  |  |  |
|  |  |  |  |  |  |  | Black African | 35.00% | 49/140 |  |  |  |
|  |  |  |  |  |  |  | Other Black | 22.22% | 14/63 |  |  |  |
|  |  |  |  |  |  |  | White | 15.45% | 17/110 |  |  |  |
|  |  |  |  |  |  |  | Mixed | 29.63% | 16/54 |  |  |  |
|  |  |  |  |  |  |  | Other | 11.30% | 26/230 |  |  |  |
|  |  |  |  |  |  |  | Not stated | 15.57% | 38/244 |  |  |  |
| BCG vaccinated | No | 18.92% | 7/37 | No | 19.67% | 107/544 |  |  |  |  |  |  |
|  | Yes | 15.96% | 30/188 | Yes | 14.16% | 16/113 |  |  |  |  |  |  |
| **Risk factor** | **Categories** | **%** | **x/n** |  |  |  |  |  |  |  |  |  |
| Time since arrival in the UK (years) | <1 | 13.89% | 5/36 |  |  |  |  |  |  |  |  |  |
|  | 1-2 | 14.29% | 15/105 |  |  |  |  |  |  |  |  |  |
|  | 3-5 | 20.45% | 18/88 |  |  |  |  |  |  |  |  |  |
| Travel to TB endemic country | No | 13.21% | 21/159 |  |  |  |  |  |  |  |  |  |
|  | Yes | 24.29% | 17/70 |  |  |  |  |  |  |  |  |  |
| TB contact | No | 15.35% | 33/215 |  |  |  |  |  |  |  |  |  |
|  | Yes | 38.46% | 5/13 |  |  |  |  |  |  |  |  |  |
| Employment status | Unemployed | 23.08% | 15/65 |  |  |  |  |  |  |  |  |  |
|  | Employed | 13.13% | 13/99 |  |  |  |  |  |  |  |  |  |
|  | Student | 15.38% | 10/65 |  |  |  |  |  |  |  |  |  |
| Alcohol | No | 17.96% | 30/167 |  |  |  |  |  |  |  |  |  |
|  | Yes | 12.90% | 8/62 |  |  |  |  |  |  |  |  |  |
| Smoker | No | 17.62% | 34/193 |  |  |  |  |  |  |  |  |  |
|  | Yes | 11.11% | 4/36 |  |  |  |  |  |  |  |  |  |
| History of | No | 16.81% | 38/226 |  |  |  |  |  |  |  |  |  |
| imprisonment | Yes | 0.00% | 0/3 |  |  |  |  |  |  |  |  |  |

* Results shown in Pareek et al 2013 (24) are for the Quantiferon IGRA test; test positivity for the TST and T.SPOT.TB tests are also provided in the paper.

** Supplementary data provided by Gareth Davies (personal communication), with additional data provided stratified by both age and sex (data not shown).

*** Reported as gender in Pareek et al 2013 (24) and sex in Pareek et al 2011 (2) and Barry et al 2023 (1).

† Testing data for 0-10 year olds were excluded because only children who were symptomatic or close contacts of active TB cases were tested.

**Table S6:** Prevalence of active TB infection stratified by risk factors, reported by included studies. Studies are arranged with equivalent/similar reported risk factors across the same row, to facilitate comparison (where studies stratify by similar but not identical risk factors, the precise risk factor used for each study is shown in brackets under “Study”).

| **Risk factor** | **Categories** | **Yield per 100,000** | **x/n** | **Categories** | **Yield per 100,000** | **x/n** |
| --- | --- | --- | --- | --- | --- | --- |
|  | **Aldridge 2016 (3)*** | | | **Crawshaw 2018 (37)** | | |
| Sex | Female | 116 | 194/167,393 |  |  |  |
|  | Male | 79 | 244/309,062 |  |  |  |
| Age (years) | 0-15 | 37 | 7/18,729 |  |  |  |
|  | 16-44 | 92 | 409/444,579 |  |  |  |
|  | 45-64 | 134 | 14/10,413 |  |  |  |
|  | ≥65 | 329 | 9/2734 |  |  |  |
| Country of | Country of screening** |  |  | Nationality |  |  |
| screening**/ | Bangladesh | 85 | 122/143,154 | Afghanistan | 0 | 0/63 |
| nationality | Burkina Faso | 0 | 0/73* | DRC | 526 | 3/570 |
|  | Cambodia | 161 | 1/621 | Eritrea | 0 | 0/59 |
|  | Côte d'Ivoire | 0 | 0/1026* | Ethiopia | 345 | 1/290 |
|  | Eritrea | 658 | 1/152 | Iran | 0 | 0/15 |
|  | Ghana | 32 | 6/18,649 | Iraq | 0 | 0/540 |
|  | Kenya | 101 | 13/12,867 | Palestine | 0 | 0/28 |
|  | Laos | 0 | 0/193* | Somalia | 356 | 2/562 |
|  | Niger | 0 | 0/36* | South Sudan | 0 | 0/40 |
|  | Pakistan | 63 | 153/243,243 | Sudan | 0 | 0/369 |
|  | Somalia | 181 | 5/2760 | Syria | 42 | 3/7195*** |
|  | Sudan | 25 | 1/4025 | Uganda | 0 | 0/2 |
|  | Togo | 0 | 0/188* | Other AFR† | 0 | 0/8 |
|  | Tanzania | 120 | 5/4166 | Other EMR† | 0 | 0/9 |
|  | Thailand | 291 | 132/45,302 |  |  |  |
| WHO TB prevalence (per 100,000) in country of screening | 40–149 | 32 | 6/18,910 |  |  |  |
|  | 150-349 | 225 | 152/67,574 |  |  |  |
|  | ≥350 | 72 | 281/389,971 |  |  |  |
| Close or household contact with an individual with TB | No | 89 | 423/475,216 |  |  |  |
|  | Yes | 1372 | 17/1239 |  |  |  |
|  | **Zenner 2023 (12)**‡ | | |  | | |
| Migrant type | UK students and workers | 54 | 572/1,056,195 |  |  |  |
|  | UK settlements and family | 108 | 624/576,485 |  |  |  |
|  | UK working holiday and others | 72 | 81/112,558 |  |  |  |

* Numerators are estimators which have been derived from the group sizes and yield per 100,000 stated in the publication. For subjects from Burkina Faso, Côte d’Ivoire, Laos, Niger and Togo, data on yield per 100,000 was blank in the reporting table, but because the derived numerators for all other country of birth strata summed to 439 (which is the total reported cases of bacteriologically confirmed tuberculosis diagnosed for the entire study population), we have assumed that yield is zero for the remaining countries. Study included in active TB prevalence estimates in forest plot is Menezes et al 2022 (11) (study years 2005-2018) but Aldridge et al 2016 (3) provides additional data on prevalence stratified by risk factors for subjects from study years 2005-2013.

** Country of screening is for migrants participating in pre-entry UK screening.

*** Yield/100,000 is stated as 41 rather than 42 in the publication.

† AFR – WHO Africa region; EMR – WHO Eastern Mediterranean Region. Other AFR included Burundi, Congo, Rwanda, Cameroon and Nigeria; Other EMR included Jordan, Lebanon, Djibouti, Yemen and Pakistan.

‡ Study included in active TB prevalence estimates in forest plot is Menezes et al 2022 (11) but Zenner et al 2023 (12) provides additional data for the same study years.

**Table S7:** Prevalence of HIV infection stratified by risk factors, reported by included studies.

| **Risk factor** | **Categories** | **%** | **x/n** | **Study** |
| --- | --- | --- | --- | --- |
| Sex | Female | 0.44% | 8/1819 | Baggaley 2025 (6) |
|  | Male | 0.52% | 9/1718 |  |
| Age (years) | 16-25 | 0.18% | 2/1109 | Baggaley 2025 (6) |
|  | 26-35 | 0.36% | 5/1397 |  |
|  | 36-45 | 0.85% | 5/588 |  |
|  | 46-55 | 1.67% | 5/299 |  |
|  | 56-65 | 0.00% | 0/140 |  |
|  | ≥66 | 0.00% | 0/12 |  |
| Nationality | Afghanistan | 0.00% | 0/56 | Crawshaw 2018 (37) |
|  | DRC | 3.57% | 18/504 |  |
|  | Eritrea | 0.00% | 0/52 |  |
|  | Ethiopia | 1.54% | 4/259 |  |
|  | Iran | 0.00% | 0/14 |  |
|  | Iraq | 0.22% | 1/462* |  |
|  | Palestine | 0.00% | 0/25 |  |
|  | Somalia | 0.60% | 3/499 |  |
|  | South Sudan | 0.00% | 0/35 |  |
|  | Sudan | 1.52% | 5/329 |  |
|  | Syria | 0.00% | 0/6245 |  |
|  | Uganda | 0.00% | 0/1 |  |
|  | Other AFR** | 0.00% | 0/8 |  |
|  | Other EMR** | 0.00% | 0/8 |  |
|  | Other WPR** | 0.00% | 0/4 |  |
| Ethnicity | Indian | 0.07% | 11444 | Baggaley 2025 (6) |
|  | Pakistani | 0.00% | 0/86 |  |
|  | Bangladeshi | 0.00% | 0/77 |  |
|  | Other Asian | 0.17% | 1/577 |  |
|  | Black African | 3.79% | 8/211 |  |
|  | Other Black | 3.70% | 4/108 |  |
|  | White | 0.00% | 0/185 |  |
|  | Mixed | 1.11% | 1/90 |  |
|  | Other | 0.00% | 0/41 |  |
|  | Not stated | 0.50% | 2/403 |  |

* Crawshaw et al (37) Table 3 states x/n as 1/462 but HIV prevalence as 0.00%. Authors were unable to verify which datum was incorrect, but other data in Table 3 suggest that the 0.00% HIV prevalence statistic is incorrect.

** AFR – WHO Africa region; EMR – WHO Eastern Mediterranean Region; WPR – WHO Western Pacific Region. Other AFR included Burundi, Congo, Rwanda, Cameroon and Nigeria; Other EMR included Jordan, Lebanon, Djibouti, Yemen and Pakistan. Other WPR included Solomon Islands, China, Taiwan or applicants with no nationality specified.

**Table S8:** Prevalence of HBV infection stratified by risk factors, reported by included studies. Studies are arranged with equivalent/similar reported risk factors across the same row, to facilitate comparison.

| **Risk factor** | **Categories** | **%** | **x/n** | **Categories** | **%** | **x/n** | **Categories** | **%** | **x/n** |
| --- | --- | --- | --- | --- | --- | --- | --- | --- | --- |
|  | **Flanagan 2019 (4)** | | | **Baggaley 2025 (6)** | | | **Uddin 2010 (5)** | | |
| Sex | Female | 0.60% | 41/6841 | Female | 1.94% | 35/1801 | Female | 0.84% | 14/1671 |
|  | Male | 1.69% | 86/5087 | Male | 4.82% | 82/1701 | Male | 1.55% | 42/2710 |
| Age (years) | 18-19 | 0.00% | 0/229 | 16-25 | 1.64% | 18/1095 |  |  |  |
|  | 20-29 | 0.81% | 18/2209* | 26-35 | 3.87% | 54/1395 | 16-29 | 0.60% | 3/503 |
|  | 30-39 | 1.10% | 34/3084 | 36-45 | 4.83% | 28/580 | 30-39 | 1.66% | 13/785 |
|  | 40-49 | 1.18% | 32/2719 | 46-55 | 4.20% | 12/286 | 40-49 | 2.00% | 17/848 |
|  | 50-59 | 1.15% | 20/1741 | 56-65 | 3.76% | 5/133 | 50-59 | 1.23% | 12/979 |
|  | 60-69 | 1.48% | 17/1147 | ≥66 | 0.00% | 0/13 | 60-69 | 0.73% | 5/689 |
|  | ≥70 | 0.75% | 6/800 |  |  |  | ≥70 | 1.04% | 6/577 |
| Ethnicity | Black | 1.37% | 9/657 | Black African | 7.52% | 16/210 |  |  |  |
|  |  |  |  | Other Black | 5.66% | 6/106 |  |  |  |
|  | Bangladeshi | 1.04% | 10/966 | Bangladeshi | 7.79% | 6/77 |  |  |  |
|  | Indian | 0.60% | 7/1173 | Indian | 1.55% | 22/1418 |  |  |  |
|  | Pakistani | 0.77% | 53/6852 | Pakistani | 2.38% | 2/84 |  |  |  |
|  | Other Asian | 2.72% | 11/405 | Other Asian | 4.53% | 26/574 |  |  |  |
|  | E European | 1.93% | 8/415 | White | 6.91% | 13/188 |  |  |  |
|  | Other | 1.98% | 29/1461 | Mixed | 6.25% | 5/80 |  |  |  |
|  |  |  |  | Other | 2.70% | 10/370 |  |  |  |
|  |  |  |  | Not stated | 2.78% | 11/395 |  |  |  |
| Length of UK stay |  |  |  |  |  |  | 0-9 | 1.71% | 15/878 |
| (years) |  |  |  |  |  |  | 10-19 | 1.37% | 8/583 |
|  |  |  |  |  |  |  | 20-29 | 1.74% | 11/633 |
|  |  |  |  |  |  |  | 30-39 | 1.03% | 9/875 |
|  |  |  |  |  |  |  | ≥40 | 0.99% | 10/1006 |
|  |  |  |  |  |  |  | Missing | 0.74% | 3/406 |
|  | **Kelly 2023 (45)** | | |  |  |  |  | | |
| Other risk factors | Blood transfusion | 0.00% | 0/75 |  |  |  |  |  |  |
|  | Surgery abroad | 0.00% | 0/327 |  |  |  |  |  |  |
|  | Dental work abroad | 0.25% | 1/403 |  |  |  |  |  |  |
|  | Vaccination** | 0.43% | 3/698 |  |  |  |  |  |  |
|  | Hepatitis vaccination** | 0.00% | 0/85 |  |  |  |  |  |  |
|  | Past history of jaundice | 0.00% | 0/73 |  |  |  |  |  |  |
|  | Family history of liver disease | 1.05% | 1/95 |  |  |  |  |  |  |
|  | Body/ear piercing | 0.34% | 3/882 |  |  |  |  |  |  |
|  | Recreational/illicit substances | 5.00% | 1/20 |  |  |  |  |  |  |
|  | Alcohol use | 0.60% | 1/166 |  |  |  |  |  |  |

* HBV prevalence in the 20-29 year category is incorrectly stated as 1.8%. It has been corrected in this table.

** ‘Vaccination’ and ‘hepatitis vaccination’ were not further defined or interpreted in the article.

**Table S9:** Prevalence of HBV infection stratified by risk factors relating to region/country of birth and origin, reported by included studies. Studies are arranged with equivalent/similar reported risk factors across the same row, to facilitate comparison (where studies stratify by similar but not identical risk factors, the precise risk factor used for each study is shown in brackets under “Study”).

| **Country/region** | **%** | **x/n** | **%** | **x/n** | **%** | **x/n** | **%** | **x/n** | **%** | **x/n** |
| --- | --- | --- | --- | --- | --- | --- | --- | --- | --- | --- |
| **COUNTRY** | **Eisen 2023 (44)**  **(country of birth)** | | **Crawshaw 2018 (37)**  **(nationality)** | | **Cochrane 2015 (34)**  **(country of birth)** | | **Mcpherson 2013 (26)**  **(country of birth)** | | **Uddin 2010 (5)***  **(country of birth)** | |
| Afghanistan | 7.85% | 15/191 | 1.75% | 1/57 |  |  |  |  |  |  |
| Albania | 0.00% | 0/71 |  |  |  |  |  |  |  |  |
| Bangladesh |  |  |  |  |  |  | 0.50% | 1/208 | 1.52% | 11/726 |
| China |  |  |  |  |  |  | 11.00% | 18/163 |  |  |
| DRC |  |  | 5.81% | 29/499 |  |  |  |  |  |  |
| Eritrea | 0.47% | 1/214 | 0.00% | 0/54 |  |  |  |  |  |  |
| Ethiopia | 8.77% | 5/57 | 4.78% | 12/251 |  |  |  |  |  |  |
| Hong Kong |  |  |  |  |  |  | 7.80% | 24/307 |  |  |
| India |  |  |  |  | 0.46% | 2/433 |  |  | 1.08% | 1/1197** |
| Iran | 0.00% | 0/95 | 0.00% | 0/14 |  |  |  |  |  |  |
| Iraq | 0.00% | 0/39 | 0.58% | 3/514 |  |  |  |  |  |  |
| Pakistan |  |  |  |  | 0.83% | 4/481 | 3.10% | 7/222 | 1.79% | 44/2458 |
| Palestine |  |  | 0.00% | 0/28 |  |  |  |  |  |  |
| Poland |  |  |  |  | 0.65% | 6/919 |  |  |  |  |
| Somalia |  |  | 3.39% | 13/384 | 2.90% | 29/1001 |  |  |  |  |
| South Sudan |  |  | 12.50% | 5/40 |  |  |  |  |  |  |
| Sudan | 7.41% | 12/162 | 5.82% | 21/361 |  |  |  |  |  |  |
| Syria | 0.00% | 0/25 | 1.46% | 102/6996 |  |  |  |  |  |  |
| Uganda |  |  | 0.00% | 0/2 |  |  |  |  |  |  |
| Vietnam | 5.43% | 5/92 |  |  |  |  | 17.40% | 4/23 |  |  |
| Other |  |  |  |  |  |  |  |  |  |  |
| Other AFR*** |  |  | 12.50% | 1/8 |  |  |  |  |  |  |
| Other EMR*** |  |  | 0.00% | 0/9 |  |  |  |  |  |  |
| Other WPR*** |  |  | 0.00% | 0/3 |  |  |  |  |  |  |
| **REGION OF BIRTH** |  | |  | |  | |  | |  |  |
| **Cochrane 2015 (34)** | | | **Mcpherson 2013 (26)** | | |  |  |  |  |  |
| **Category** | **%** | **x/n** | **Category** | | **%** | **x/n** |  |  |  |  |
| Africa | 2.49% | 49/1965 |  |  |  |  |  |  |  |  |
| Eastern Africa | 2.33% | 32/1372 |  |  |  |  |  |  |  |  |
| Middle Africa | 7.84% | 4/51 |  |  |  |  |  |  |  |  |
| Northern Africa | 1.23% | 1/81 |  |  |  |  |  |  |  |  |
| Southern Africa | 0.00% | 0/140 |  |  |  |  |  |  |  |  |
| Western Africa | 4.05% | 13/321 |  |  |  |  |  |  |  |  |
| Asia and Pacific | 1.88% | 36/1917 |  |  |  |  |  |  |  |  |
| Central Asia | 0.00% | 0/7 |  |  |  |  |  |  |  |  |
| **Category** | **%** | **x/n** | **Category** | | **%** | **x/n** |  |  |  |  |
| Eastern Asia | 9.95% | 20/201 | British-Chinese | | 9.04% | 48/531 |  |  |  |  |
| Southern Asia | 0.74% | 9/1219 | South Asian | | 2.00% | 9/449 |  |  |  |  |
| South Eastern Asia | 3.07% | 8/261 | Other country, British-Chinese subjects | | 5.30% | 2/38 |  |  |  |  |
| Western Asia | 0.46% | 1/219 | Other country, South Asian subjects | | 5.20% | 1/19 |  |  |  |  |
| Pacific Islands | 0.00% | 0/10 |  |  |  |  |  |  |  |  |
| Latin America and Caribbean | 0.60% | 2/333 |  |  |  |  |  |  |  |  |
| Caribbean | 0.93% | 2/215 |  |  |  |  |  |  |  |  |
| Central America | 0.00% | 0/14 |  |  |  |  |  |  |  |  |
| South America | 0.00% | 0/104 |  |  |  |  |  |  |  |  |
| Eastern and South Europe | 0.80% | 13/1625 |  |  |  |  |  |  |  |  |
| Eastern Europe | 0.86% | 11/1276 |  |  |  |  |  |  |  |  |
| South Europe | 0.29% | 1/349 |  |  |  |  |  |  |  |  |

* Uddin et al 2010 (5) additionally provides within-country estimates for Pakistan: 1.53% (16/1049) for Punjabi region, 2.46% (14/570) for Kashmir region, 2.93% (7/239) for other region of Pakistan and 1.17% (7/600) for Pakistani subjects of unknown region.

** Includes people born in India, but also people who originated in India and came to the UK via Africa (Kenya N = 131, Tanzania N = 23 or Uganda N = 69).

*** AFR – WHO Africa region; EMR – WHO Eastern Mediterranean Region; WPR – WHO Western Pacific Region. Other AFR included Burundi, Congo, Rwanda, Cameroon and Nigeria; Other EMR included Jordan, Lebanon, Djibouti, Yemen and Pakistan. Other WPR included Solomon Islands, China, Taiwan or applicants with no nationality specified.

**Table S10:** Prevalence of current HCV infection (HCV RNA test) stratified by risk factors, reported by included studies. Studies are arranged with equivalent/similar reported risk factors across the same row, to facilitate comparison (where studies stratify by similar but not identical risk factors, the precise risk factor used for each study is shown in brackets under “Study”).

| **Risk factor** | **Categories** | **%, x/n** | **Categories** | **%, x/n** | **Categories** | **%, x/n** | **Categories** | **%, x/n** |
| --- | --- | --- | --- | --- | --- | --- | --- | --- |
|  | **Flanagan 2019 (4)** | | **Uddin 2010 (5)** | | **Baggaley 2025 (6)** | |  | |
| Sex | Female | 0.29%, 20/6841 | Female | 1.56%, 26/1671 | Female | 0.11%, 2/1759 |  |  |
|  | Male | 0.31%, 16/5087 | Male | 1.73%, 47/2710 | Male | 0.24%, 4/1643 |  |  |
| Age (years) | 18-19 | 0.00%, 0/229 |  |  | 16-25 | 0.00%, 0/1068 |  |  |
|  | 20-29 | 0.23%, 5/2209 | 16-29 | 2.39%, 12/503 | 26-35 | 0.15%, 2/1347 |  |  |
|  | 30-39 | 0.52%, 16/3084 | 30-39 | 2.42%, 19/785 | 36-45 | 0.18%, 1/565 |  |  |
|  | 40-49 | 0.26%, 7/2719 | 40-49 | 1.53%, 13/848 | 46-55 | 1.07%, 3/280 |  |  |
|  | 50-59 | 0.29%, 5/1741 | 50-59 | 1.33%, 13/979 | 56-65 | 0.00%, 0/130 |  |  |
|  | 60-69 | 0.09%, 1/1147 | 60-69 | 0.87%, 6/689 | ≥66 | 0.00%, 0/12 |  |  |
|  | ≥70 | 0.25%, 2/800 | ≥70 | 1.73%, 10/577 |  |  |  |  |
|  | **Flanagan 2019 (4) – ethnicity** | | **Uddin 2010 (5)* – country of birth** | | **Baggaley 2025 (6) – ethnicity** | | **Crawshaw 2018 (37) – nationality** | |
| Ethnicity / country of birth / nationality | Black | 0.00%, 0/657 |  |  | Black African | 0.00%, 0/196 | Afghanistan | 1.75%, 1/57 |
|  |  |  |  |  | Other Black | 0.00% , 0/98 | DRC | 0.80%, 4/499 |
|  | Bangladeshi | 0.00%, 0/966 | Bangladesh | 0.55%, 4/726 | Bangladeshi | 0.00%, 0/76 | Eritrea | 0.00%, 0/54 |
|  | Indian | 0.17%, 2/1173 | India | 0.17%, 2/1197** | Indian | 0.22%, 3/1392 | Ethiopia | 0.40%, 1/250 |
|  | Pakistani | 0.47%, 32/6852 | Pakistan | 2.73%, 67/2458 | Pakistani | 0.00%, 0/79 | Iran | 7.14%, 1/14 |
|  | Other Asian | 0.00%, 0/405 |  |  | Other Asian | 0.18%, 1/561 | Iraq | 0.00%, 0/517 |
|  | E European | 0.48%, 2/415 |  |  | White | 0.00%, 0/181 | Palestine | 0.00%, 0/28 |
|  | Other | 0.00%, 0/1461 |  |  | Mixed | 0.00%, 0/75 | Somalia | 0.26%, 1/382 |
|  |  |  |  |  | Other | 0.56%, 2/357 | South Sudan | 0.00%, 0/40 |
|  |  |  |  |  | Not stated | 0.00%, 0/387 | Sudan | 0.28%, 1/361 |
|  |  |  |  |  |  |  | Syria | 0.41%, 29/6994 |
|  |  |  |  |  |  |  | Uganda | 0.00%, 0/2 |
|  |  |  |  |  |  |  | Other AFR*** | 0.00%, 0/8 |
|  |  |  |  |  |  |  | Other EMR*** | 0.00%, 0/9 |
|  |  |  |  |  |  |  | Other WPR*** | 0.00%, 0/3 |
| Length of UK stay (years) |  |  | 0-9 | 3.76%, 33/878 |  |  |  |  |
|  |  |  | 10-19 | 1.72%, 10/583 |  |  |  |  |
|  |  |  | 20-29 | 0.95%, 6/633 |  |  |  |  |
|  |  |  | 30-39 | 0.69%, 6/875 |  |  |  |  |
|  |  |  | ≥40 | 0.89%, 9/1006 |  |  |  |  |
|  |  |  | Missing | 2.22%, 9/406 |  |  |  |  |

* Uddin et al 2010 (5) additionally provides within-country estimates for Pakistan: 3.72% (39/1049) for Punjabi region, 0.53% (3/570) for Kashmir region, 0.84% (2/239) for other region of Pakistan and 3.83% (23/600) for Pakistani subjects of unknown region.

** Includes people born in India, but also people who originated in India and came to the UK via Africa (Kenya N = 131, Tanzania N = 23 or Uganda N = 69).

*** AFR – WHO Africa region; EMR – WHO Eastern Mediterranean Region; WPR – WHO Western Pacific Region. Other AFR included Burundi, Congo, Rwanda, Cameroon and Nigeria; Other EMR included Jordan, Lebanon, Djibouti, Yemen and Pakistan. Other WPR included Solomon Islands, China, Taiwan or applicants with no nationality specified.

**Table S11:** Prevalence of history of HCV infection (HCV antibody test) stratified by risk factors, reported by included studies. Studies are arranged with equivalent/similar reported risk factors across the same row, to facilitate comparison (where studies stratify by similar but not identical risk factors, the precise risk factor used for each study is shown in brackets under “Study”).

| **Risk factor** | **Categories** | **%, x/n** | **Study** | **Categories** | **%, x/n** | **Study** |
| --- | --- | --- | --- | --- | --- | --- |
|  | **STUDY 1** | | | **STUDY 2** | | |
| Sex | Female | 0.92%, 63/6841 | Flanagan 2019 (4) |  |  |  |
|  | Male | 0.94%, 48/5087 |  |  |  |  |
| Age (years) | 18-19 | 0.00%, 0/229 | Flanagan 2019 (4) | <21 | 0.00%, 0/171 | Cortina-Borja 2016 (48)* |
|  | 20-29 | 0.36%, 8/2209 |  | 21-25 | 0.08%, 1/1297 |  |
|  | 30-39 | 1.13%, 35/3084 |  | 26-30 | 0.39%, 10/2583 |  |
|  | 40-49 | 1.25%, 34/2719 |  | 31-35 | 0.16%, 3/1857 |  |
|  | 50-59 | 1.09%, 19/1741 |  | >35 | 0.44%, 6/1369 |  |
|  | 60-69 | 0.70%, 8/1147 |  |  |  |  |
|  | ≥70 | 0.88%, 7/800 |  |  |  |  |
| Ethnicity/country of birth | Black | 0.30%, 2/657 | Flanagan 2019 (4) |  |  | Mcpherson 2013 (26)** |
|  | Bangladeshi | 0.31%, 3/966 | (ethnicity) | Bangladesh | 0.00%, 0/208 | (country of birth) |
|  | Indian | 0.34%, 4/1173 |  |  |  |  |
|  | Pakistani | 1.30%, 89/6852 |  | Pakistan | 1.80%, 4/222 |  |
|  |  |  |  | South Asian | 0.89%, 4/449 |  |
|  | Other Asian | 0.25%, 1/405 |  |  |  |  |
|  | E European | 0.96%, 4/415 |  |  |  |  |
|  | Other | 0.55%, 8/1461 |  |  |  |  |
| Region of birth | Africa | 0.03%, 1/3189 | Cortina-Borja 2016 (48)*** |  |  |  |
|  | Northern Africa | 0.00%, 0/304 |  |  |  |  |
|  | Western Africa | 0.00%, 0/1154 |  |  |  |  |
|  | Central Africa | 0.40%, 1/253 |  |  |  |  |
|  | Eastern Africa | 0.00%, 0/1258 |  |  |  |  |
|  | Southern Africa | 0.00%, 0/220 |  |  |  |  |
|  | Europe (excluding UK) | 0.27%, 12/4492 |  |  |  |  |
|  | Northern Europe | 0.00%, 0/332 |  |  |  |  |
|  | Western Europe | 0.00%, 0/526 |  |  |  |  |
|  | Eastern Europe | 0.37%, 11/3008 |  |  |  |  |
|  | Southern Europe | 0.16%, 1/626 |  |  |  |  |
|  | Americas | 0.00%, 0/835 |  |  |  |  |
|  | North America | 0.00%, 0/286 |  |  |  |  |
|  | Central America & Caribbean | 0.00%, 0/275 |  |  |  |  |
|  | South America | 0.00%, 0/274 |  |  |  |  |
|  | Asia-Pacific | 0.17%, 10/5853 |  |  |  |  |
|  | Western Asia | 0.00%, 0/747 |  |  |  |  |
|  | Central Asia | 0.22%, 1/458 |  |  |  |  |
|  | Southern Asia | 0.16%, 6/3697 |  |  |  |  |
|  | South Eastern Asia | 0.26%, 1/389 |  |  |  |  |
|  | Eastern Asia | 0.30%, 1/328 |  |  |  |  |
|  | Oceania | 0.43%, 1/234 |  |  |  |  |
|  | Not known | 0.26%, 4/1532 |  |  |  |  |
| Other risk factors | Blood transfusion | 0.00%, 0/75 | Kelly 2023 (45) |  |  |  |
|  | Surgery abroad | 0.31%, 1/327 |  |  |  |  |
|  | Dental work abroad | 0.25%, 1/403 |  |  |  |  |
|  | Vaccination | 0.29%, 2/698 |  |  |  |  |
|  | Hepatitis vaccination | 0.00%, 0/85 |  |  |  |  |
|  | Past history of jaundice | 0.00%, 0/73 |  |  |  |  |
|  | Family history of liver disease | 0.00%, 0/95 |  |  |  |  |
|  | Body/ear piercing | 0.45%, 4/882 |  |  |  |  |
|  | Recreational/illicit substances | 5.00%, 1/20 |  |  |  |  |
|  | Alcohol use | 0.60%, 1/166 |  |  |  |  |

* Includes women from Eastern Europe and Asia Pacific regions only. Results shown in this table are for these regions combined, but prevalence stratified by both age and region are presented in Table 2 of Cortina-Borja et al (48).

** Only study participants of South Asian world region of origin were tested for past HCV infection.

*** Maternal region of birth: residual neonatal dried blood spot samples routinely collected for metabolic newborn screening.

**References**

1. Barry SM, Davies G, Barry TD, Evans J, Backx M, Brouns M, et al. Outcomes from a national screening program for Ukrainian refugees at risk of drug resistant tuberculosis in Wales. Thorax. 2023;79(1):86-9.

2. Pareek M, Watson JP, Ormerod LP, Kon OM, Woltmann G, White PJ, et al. Screening of immigrants in the UK for imported latent tuberculosis: a multicentre cohort study and cost-effectiveness analysis. Lancet Infect Dis. 2011;11(6):435-44.

3. Aldridge RW, Zenner D, White PJ, Muzyamba MC, Loutet M, Dhavan P, et al. Prevalence of and risk factors for active tuberculosis in migrants screened before entry to the UK: a population-based cross-sectional study. Lancet Infect Dis. 2016;16(8):962-70.

4. Flanagan S, Kunkel J, Appleby V, Eldridge SE, Ismail S, Moreea S, et al. Case finding and therapy for chronic viral hepatitis in primary care (HepFREE): a cluster-randomised controlled trial. Lancet Gastroenterol Hepatol. 2019;4(1):32-44.

5. Uddin G, Shoeb D, Solaiman S, Marley R, Gore C, Ramsay M, et al. Prevalence of chronic viral hepatitis in people of south Asian ethnicity living in England: the prevalence cannot necessarily be predicted from the prevalence in the country of origin. J Viral Hepat. 2010;17(5):327-35.

6. Baggaley RF, Martin CA, Eborall HC, Gohar M, Aziz K, Fahad M, et al. Community-based testing of migrants for infectious diseases (COMBAT-ID): observational cohort study measuring the effectiveness of routine testing for infectious diseases among migrants attending primary care. eClinicalMedicine. 2025;Online first103253 May 30, 2025.

7. NICE. National Institute for Health and Care Excellence (NICE) and Public Health England (PHE). HIV testing: increasing uptake among people who may have undiagnosed HIV. NICE guideline NG60. Published 1 December 2016. Available from: <www.nice.org.uk/guidance/ng60> Accessed 25 January 2026. 2016.

8. BHIVA. British HIV Association (BHIVA)/British Association for Sexual Health and HIV (BASHH)/British Infection Association (BIA). Adult HIV Testing Guidelines 2020. Available from: <https://bhiva.org/clinical-guideline/hiv-testing-guidelines/> Accessed 25 January 2026. 2020.

9. WHO. World Health Organization (WHO). WHO Guidelines on Hepatitis B and C Testing. Geneva: World Health Organization. Table 1: Summary of recommendations on testing for chronic hepatitis B and C virus infection. Available from: <https://www.who.int/publications/i/item/9789241549981> Accessed 24 January 2026. 2017.

10. UKHSA. UK Health Security Agency (UKHSA). Tuberculosis (TB): migrant health guide. Available from: <https://www.gov.uk/guidance/tuberculosis-tb-migrant-health-guide> Accessed 25 January 2026. 2014.

11. Menezes D, Zenner D, Aldridge R, Anderson SR, de Vries G, Erkens C, et al. Country differences and determinants of yield in programmatic migrant TB screening in four European countries. Int J Tuberc Lung Dis. 2022;26(10):942-8.

12. Zenner D, Brals D, Nederby-Ohd J, Menezes D, Aldridge R, Anderson SR, et al. Drivers determining tuberculosis disease screening yield in four European screening programmes: a comparative analysis. Eur Respir J. 2023;62(4).

13. Severi E, Maguire H, Ihekweazu C, Bickler G, Abubakar I. Outcomes analysis of new entrant screening for active tuberculosis in Heathrow and Gatwick airports, United Kingdom 2009/2010. BMC Infect Dis. 2016;16:178.

14. WHO. World Health Organization TB profiles. Available from: <https://worldhealthorg.shinyapps.io/tb_profiles/?_inputs_&tab=%22tables%22&lan=%22EN%22&iso2=%22RU%22&entity_type=%22country%22> Accessed 11 June 2025.

15. WHO. World Health Organization. The Global Health Observatory. Available from: <https://www.who.int/data/gho/data/indicators/indicator-details/GHO/prevalence-of-hiv-among-adults-aged-15-to-49-(->) Accessed 11 June 2025.

16. UNAIDS. UNAIDS country summaries. Available from: <https://www.unaids.org/en/regionscountries/countries> Accessed 11 June 2025.

17. The Coalition for Global Hepatitis Elimination. Data and Profiles. Available from: <https://www.globalhep.org/data-profiles> Accessed 11 June 2025.

18. ONS. Office for National Statistics Dataset: Population by country of birth and nationality. Available from: <https://www.ons.gov.uk/peoplepopulationandcommunity/populationandmigration/internationalmigration/datasets/populationoftheunitedkingdombycountryofbirthandnationality> Accessed 11 June 2025.

19. UNAIDS. UNAIDS estimates, Joint United Nations Programme on HIV/AIDS ( UNAIDS ), uri: aidsinfo.unaids.org, publisher: UNAIDS, data accessed via The World Bank. Available from: <https://data.worldbank.org/indicator/SH.DYN.AIDS.ZS> Date accessed: 27 January 2026.

20. Coalition for Global Hepatitis Elimination and The Task Force For Global Health. Global Statistics. Available from: <https://www.globalhep.org/data-profiles> Accessed 27 January 2026.

21. Harling R, Pearce M, Chandrakumar M, Mueller K, Hayward A. Tuberculosis screening of asylum seekers: 1 years' experience at the Dover Induction Centres. Public Health. 2007;121(11):822-7.

22. Burns FM, Mercer CH, Evans AR, Gerry CJ, Mole R, Hart GJ. Increased attendances of people of eastern European origin at sexual health services in London. Sex Transm Infect. 2009;85(1):75-8.

23. Platt L, Grenfell P, Bonell C, Creighton S, Wellings K, Parry J, et al. Risk of sexually transmitted infections and violence among indoor-working female sex workers in London: the effect of migration from Eastern Europe. Sex Transm Infect. 2011;87(5):377-84.

24. Pareek M, Bond M, Shorey J, Seneviratne S, Guy M, White P, et al. Community-based evaluation of immigrant tuberculosis screening using interferon gamma release assays and tuberculin skin testing: observational study and economic analysis. Thorax. 2013;68(3):230-9.

25. Vedio AB, Ellam H, Rayner F, Stone B, Kudesia G, McKendrick MW, et al. Hepatitis B: report of prevalence and access to healthcare among Chinese residents in Sheffield UK. J Infect Public Health. 2013;6(6):448-55.

26. McPherson S, Valappil M, Moses SE, Eltringham G, Miller C, Baxter K, et al. Targeted case finding for hepatitis B using dry blood spot testing in the British-Chinese and South Asian populations of the North-East of England. J Viral Hepat. 2013;20(9):638-44.

27. O'Leary MC, Sarwar M, Hutchinson SJ, Weir A, Schofield J, McLeod A, et al. The prevalence of hepatitis C virus among people of South Asian origin in Glasgow - results from a community based survey and laboratory surveillance. Travel Med Infect Dis. 2013;11(5):301-9.

28. Balogun MA, Parry JV, Mutton K, Okolo C, Benons L, Baxendale H, et al. Hepatitis B virus transmission in pre-adolescent schoolchildren in four multi-ethnic areas of England. Epidemiol Infect. 2013;141(5):916-25.

29. Sidebottom M, Street E. HIV testing in an asylum seeker initial accommodation centre. Abstract P252. . HIV Medicine. 2014;15:97.

30. O'Shea MK, Fletcher TE, Beeching NJ, Dedicoat M, Spence D, McShane H, et al. Tuberculin skin testing and treatment modulates interferon-gamma release assay results for latent tuberculosis in migrants. PLoS One. 2014;9(5):e97366.

31. Mc Grath-Lone L, Marsh K, Hughes G, Ward H. The sexual health of female sex workers compared with other women in England: analysis of cross-sectional data from genitourinary medicine clinics. Sex Transm Infect. 2014;90(4):344-50.

32. Mc Grath-Lone L, Marsh K, Hughes G, Ward H. The sexual health of male sex workers in England: analysis of cross-sectional data from genitourinary medicine clinics. Sex Transm Infect. 2014;90(1):38-40.

33. Hargreaves S, Seedat F, Car J, Escombe R, Hasan S, Eliahoo J, et al. Screening for latent TB, HIV, and hepatitis B/C in new migrants in a high prevalence area of London, UK: a cross-sectional study. BMC Infect Dis. 2014;14:657.

34. Cochrane A, Evlampidou I, Irish C, Ingle SM, Hickman M. Hepatitis B infection prevalence by country of birth in migrant populations in a large UK city. J Clin Virol. 2015;68:79-82.

35. Evlampidou I, Hickman M, Irish C, Young N, Oliver I, Gillett S, et al. Low hepatitis B testing among migrants: a cross-sectional study in a UK city. Br J Gen Pract. 2016;66(647):e382-91.

36. Usdin M, Dedicoat M, Gajraj R, Harrison P, Kaur H, Duffield K, et al. Latent tuberculous screening of recent migrants attending language classes: a cohort study and cost analysis. Int J Tuberc Lung Dis. 2017;21(2):175-80.

37. Crawshaw AF, Pareek M, Were J, Schillinger S, Gorbacheva O, Wickramage KP, et al. Infectious disease testing of UK-bound refugees: a population-based, cross-sectional study. BMC Med. 2018;16(1):143.

38. Abubakar I, Drobniewski F, Southern J, Sitch AJ, Jackson C, Lipman M, et al. Prognostic value of interferon-gamma release assays and tuberculin skin test in predicting the development of active tuberculosis (UK PREDICT TB): a prospective cohort study. Lancet Infect Dis. 2018;18(10):1077-87.

39. Berrocal-Almanza LC, Harris R, Lalor MK, Muzyamba MC, Were J, O'Connell AM, et al. Effectiveness of pre-entry active tuberculosis and post-entry latent tuberculosis screening in new entrants to the UK: a retrospective, population-based cohort study. Lancet Infect Dis. 2019;19(11):1191-201.

40. Hargreaves S, Nellums LB, Johnson C, Goldberg J, Pantelidis P, Rahman A, et al. Delivering multi-disease screening to migrants for latent TB and blood-borne viruses in an emergency department setting: A feasibility study. Travel Med Infect Dis. 2020;36:101611.

41. Kelly C, Pericleous M, Ahmed A, Vandrevala T, Hendy J, Shafi S, et al. Improving uptake of hepatitis B and hepatitis C testing in South Asian migrants in community and faith settings using educational interventions-A prospective descriptive study. Int J Infect Dis. 2020;100:264-72.

42. Pinto AC, Seery P, Foster C. Evaluating a clinic for unaccompanied asylum seeking children (UASC): the importance of infectious disease screening and a holistic approach to care. Abstract 769. Arch Dis Child. 2022;107:A327.

43. Berrocal-Almanza LC, Harris RJ, Collin SM, Muzyamba MC, Conroy OD, Mirza A, et al. Effectiveness of nationwide programmatic testing and treatment for latent tuberculosis infection in migrants in England: a retrospective, population-based cohort study. Lancet Public Health. 2022;7(4):e305-e15.

44. Eisen S, Williams B, Cohen J. Infections in Asymptomatic Unaccompanied Asylum-seeking Children in London 2016-2022. Pediatr Infect Dis J. 2023;42(12):1051-5.

45. Kelly C, Mathew S, Petrova M, Shafi S, Nicholls M, Dar O, et al. Exploring awareness and prevalence of chronic viral hepatitis in a UK based Nepali population - lessons learned for future models in engaging migrant communities. Clin Med (Lond). 2023;23(6):563-70.

46. Amrose A, Ramshaw S, Dwyer E, Bull L. Prevalence and presentations of HIV in asylum seekers in the London borough of Hounslow. Abstract P089. British HIV Association (BHIVA) Spring Conference 2023. HIV Medicine. 2023;24:78.

47. Martyn E, O'Regan S, Harris P, Leonard M, Veitch M, Sultan B, et al. Hepatitis B virus (HBV) screening, linkage and retention-in-care in inclusion health populations: Evaluation of an outreach screening programme in London. J Infect. 2024;88(2):167-72.

48. Cortina-Borja M, Williams D, Peckham CS, Bailey H, Thorne C. Hepatitis C virus seroprevalence in pregnant women delivering live-born infants in North Thames, England in 2012. Epidemiol Infect. 2016;144(3):627-34.

49. Joanna Briggs Institute Critical Appraisal Tools for Prevalence Studies. Available from: <https://jbi.global/critical-appraisal-tools> Accessed 22 July 2024. 2020.
